# Annual Protist Community Dynamics in a Freshwater Ecosystem Undergoing Contrasted Climatic Conditions: The Saint-Charles River (Canada)

**DOI:** 10.3389/fmicb.2019.02359

**Published:** 2019-10-16

**Authors:** Perrine Cruaud, Adrien Vigneron, Marie-Stéphanie Fradette, Caetano C. Dorea, Alexander I. Culley, Manuel J. Rodriguez, Steve J. Charette

**Affiliations:** ^1^Institut de Biologie Intégrative et des Systèmes, Université Laval, Québec City, QC, Canada; ^2^Département de Biochimie, de Microbiologie et de Bio-Informatique, Faculté des Sciences et de Génie, Université Laval, Québec City, QC, Canada; ^3^CRAD, Université Laval, Québec City, QC, Canada; ^4^Centre D’Études Nordiques, Université Laval, Québec City, QC, Canada; ^5^Département de Biologie, Université Laval, Québec City, QC, Canada; ^6^Department of Civil Engineering, University of Victoria, Victoria, BC, Canada; ^7^Groupe de Recherche en Ecologie Buccale, Faculté de Médecine Dentaire, Université Laval, Québec City, QC, Canada; ^8^École Supérieure D’aménagement du Territoire et de Développement Régional (ESAD), Université Laval, Québec City, QC, Canada; ^9^Centre de Recherche de l’Institut Universitaire de Cardiologie et de Pneumologie de Québec, Québec City, QC, Canada

**Keywords:** microbial eukaryotes, protists, freshwater, river, seasonal cycles, winter, bacterial communities

## Abstract

Protists are key stone components of aquatic ecosystems, sustaining primary productivity and aquatic food webs. However, their diversity, ecology and structuring factors shaping their temporal distribution remain strongly misunderstood in freshwaters. Using high-throughput sequencing on water samples collected over 16 different months (including two summer and two winter periods), combined with geochemical measurements and climate monitoring, we comprehensively determined the pico- and nanoeukaryotic community composition and dynamics in a Canadian river undergoing prolonged ice-cover winters. Our analysis revealed a large protist diversity in this fluctuating ecosystem and clear seasonal patterns demonstrating a direct and/or indirect selective role of abiotic factors, such as water temperature or nitrogen concentrations, in structuring the eukaryotic microbial community. Nonetheless, our results also revealed that primary productivity, predatory as well as parasitism lifestyles, inferred from fine phylogenetic placements, remained potentially present over the annual cycle, despite the large seasonal fluctuations and the remodeling of the community composition under ice. In addition, potential interplays with the bacterial community composition were identified supporting a possible contribution of the bacterial community to the temporal dynamics of the protist community structure. Our results illustrate the complexity of the eukaryotic microbial community and provide a substantive and useful dataset to better understand the global freshwater ecosystem functioning.

## Introduction

Eukaryotic microorganisms are widespread, diverse and involved in global functioning of ecosystems (Sherr and Sherr, 1988; Caron et al., 2009; Worden et al., 2015; Debroas et al., 2017). As primary producers, global consumers or prey for larger microbial grazers and metazoans, they represent a key link in aquatic food webs (Sherr and Sherr, 1988; Arndt, 1993; Premke and Arndt, 2000; Šlapeta et al., 2005). However, they have received less attention than prokaryotes in microbial ecology (Simon et al., 2015; Debroas et al., 2017) and their diversity has been generally less investigated in freshwaters than in oceans (Nolte et al., 2010; Debroas et al., 2017). Therefore, there is a gap in the current knowledge of the distribution, biodiversity and temporal dynamics of these microorganisms in freshwater environments (Triadó-Margarit and Casamayor, 2012).

From autotrophy (photosynthesizers) to heterotrophy (predators feeding on other eukaryotic or prokaryotic organisms, decomposers, saprophytes, and parasites of other organisms), through mixotrophy, protists cover multiple ecological roles in ecosystems (Arndt et al., 2000; Zubkov and Tarran, 2008). While it was commonly accepted that some eukaryotic phyla were strictly photosynthetic or phagotrophic, numerous studies have demonstrated that many groups are actually more flexible in their nutritional capabilities than initially thought (Laval-Peuto and Febvre, 1986; McManus and Fuhrman, 1986; Stoecker and Silver, 1987; McManus et al., 2018). For example, photosynthetic capability *via* endosymbiotic associations or chloroplast retention (uptake of chloroplast from their prey, or kleptoplasty) has been observed in a broad range of eukaryotic lineages, such as ciliates, that were previously associated with a phagotrophic non-photosynthetic lifestyle (Stoecker and Silver, 1987; Dolan, 1992; Johnson, 2011; McManus et al., 2018) or dinoflagellates (Minnhagen et al., 2008; Hansen et al., 2016). Likewise, the loss of chloroplasts by lineages within commonly accepted photosynthetic groups has also been detected, such as the *Paraphysomonas*-clade within *Chrysophyceae* (Stramenopiles) (Caron et al., 1999; Boenigk et al., 2005; Scoble and Cavalier-Smith, 2014; Grossmann et al., 2016). Mixotrophy, defined as the combination of phagotrophy and photosynthesis in an individual cell, is increasingly recognized as an important trophic mode among aquatic eukaryotes and is likely the rule rather than the exception in microbial food webs (Caron et al., 1999; Stoecker et al., 2017; McManus et al., 2018; Stoecker and Lavrentyev, 2018). In addition, bacterivorous eukaryotes do not feed equally on all microorganisms. Some of them are considered generalists, feeding on a broad range of prey, while others are specialists, feeding on a narrow range of species (Haraguchi, 2018). Thus, each phagotrophic eukaryotic species might have different prey preferences affecting the microbial assemblage differently (Massana et al., 2009). Moreover, growth, grazing and photosynthesis rates as well as prey preferences could be greatly modulated depending on environmental factors such as temperature (Wilken et al., 2013), light (Millette et al., 2017) and nutrient availability (Urabe et al., 1999; Kamjunke et al., 2006; Stoecker and Lavrentyev, 2018) or presence/absence of some microbial lineages (Terrado et al., 2017), adding a supplementary layer of complexity in microeukaryote ecology.

While amplicon sequencing and “omic” analyses allow the identification of prokaryotic microorganisms as well as their functional and nutritional capabilities which might explain temporal and spatial dynamics (Logares et al., 2014; Lima-Mendez et al., 2015; Salcher et al., 2015; Samad and Bertilsson, 2017; Vigneron et al., 2018, 2019; Cruaud et al., 2019), these analyses do not yet allow as easily the assignment of functional roles to complex eukaryotic communities. Over the past few years, increasing efforts have been made to understand their ecological roles using extensive genome-level description and single-cell sequencing revealing genomic elements associated with bacterivorous or photosynthetic lifestyles (Roy et al., 2014; Seeleuthner et al., 2018). However, these studies are still scarce and challenged by the high eukaryotic genome size that leads to highly fragmented assemblies and incomplete genomes (Seeleuthner et al., 2018). Therefore, the understanding of the temporal dynamics of eukaryotic microorganisms and the underlying environmental drivers remains a real challenge in environmental microbial ecology.

Previous studies on freshwater environments showed that seasonal cycles characterized by both abiotic and biotic disturbances may influence the protist community composition and more broadly control the structure of all biota (Kalff and Knoechel, 1978; Carrias et al., 1998; Lepère et al., 2006; Zhao et al., 2011; Sommer et al., 2012; Jones et al., 2013; Simon et al., 2015). For its part, the Plankton Ecology Group (PEG) model propose a standard template to describe the successive stages of the phytoplankton and zooplankton biomasses in lake ecosystems. It also indicates potential factors driving these seasonal successions and emphasizes that further investigations at finer taxonomical level are required to complete this model (Sommer et al., 1986, 2012). Study of the temporal dynamics of eukaryotic microbial community composition in freshwater ecosystems experiencing large fluctuations in environmental parameters, that lead to marked changes in populations, can therefore contribute to a better understanding of the mechanisms driving the dynamics of these communities.

The Saint-Charles River, located in Quebec, Canada, experiences contrasted seasons with long freezing winters and relatively short temperate summers (Cruaud et al., 2019) and might therefore represent an interesting environment to investigate microeukaryotic ecology in freshwaters. The harsh climatic conditions of the region have been shown to induce an annual cycle of bacterial communities in this river with rapid successions of bacterial lineages (Cruaud et al., 2019). Bacterial communities detected during the warm season (WS) were typical of other freshwater ecosystems with high proportions of *Limnohabitans* and *Actinobacteria*, such as *Sporichthyaceae* hgcI/acI lineages, which are frequently reported as prey for eukaryotes (Grujcic et al., 2018; Piwosz et al., 2018; Šimek et al., 2018). By contrast, the bacterial communities changed during winter, with a notable increase in proportions of potential gammaproteobacterial methanotrophs (Cruaud et al., 2019), revealing a deep modification of the microbial ecosystem, with potential consequences on aquatic food webs.

The present study is therefore the eukaryotic counterpart of the bacterial community analysis conducted in the Saint-Charles River (Cruaud et al., 2019) and aim to better understand the dynamics of eukaryotic microbial communities in freshwater ecosystems and the underlying mechanisms of the global functioning of this river.

With these objectives, we have monitored the changes in the community structure of pico- and nanoplanktonic protists (i.e., small microbial eukaryotes of 0.22–20 μm diameter) in the Saint-Charles River water samples collected over 16 different months (including two summer and two winter periods) using massive sequencing of the 18S rRNA gene (rDNA). We then examined these results in the light of environmental parameters, such as geochemical measurements and climate monitoring. After an overall analysis of the entire protist community structure, we focused on a subset of 25 dominant eukaryotic OTUs that explained most of the community variation across samples. We investigated in detail the dynamics and potential trophic modes of these protists using phylogenetic analyses and looked for interplays between eukaryotic, bacterial microbial communities and environmental parameters.

## Materials and Methods

### Sampling Sites and Methods

**FIGURE 1:**
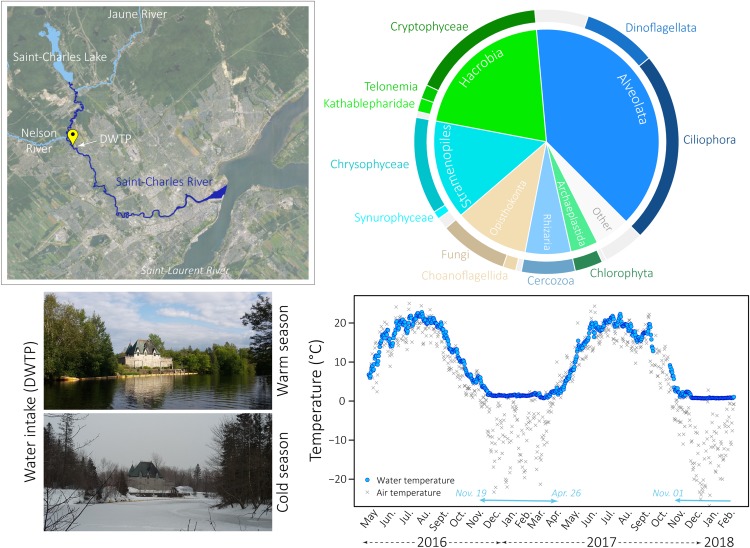
Global eukaryotic microbial community in the Saint-Charles River. Top left: map of the Saint-Charles River showing the principal tributaries (Saint-Charles Lake and Jaune and Nelson Rivers) and the sampling location (DWTP) (QGIS software, v. 2.8.6-Wien, Geoindex + platform with geospatial data from Ville de Quebec held by the Library of the Université Laval). Bottom left: pictures of the Saint-Charles River taken at the sampling location during the warm and the cold season. Bottom right: daily measurements of air and water temperatures at the water intake from May 2016 to February 2018. Turquoise arrows at the bottom of the graph indicate the presence of snow on the ground. Top right: pie charts representing the average proportion of the main microbial eukaryotic communities detected in all samples.

The Saint-Charles River, a major drinking water source in the Quebec city region, is evenly fed by the Saint-Charles Lake and two main tributaries (Jaune and Nelson Rivers) (Figure 1). The river flow of the Saint-Charles River is mainly controlled by flood gates at the Saint-Charles Lake dam to ensure adequate supply for the Loretteville Drinking Water Treatment Plant (DWTP – Quebec city, 11 km downstream of the dam) and a minimum ecological flow in the river. The Saint-Charles Lake is stratified during summer and winter and mixed during fall and spring turnovers (Boulé et al., 2010). The lake is ice-covered during winter and usually ice-free after mid-April or early May, before the spring turnover (APEL, 2015). The Saint-Charles River water samples were collected weekly or bimonthly at the raw water intake of the Loretteville DWTP (Figure 1) from May 2016 to June 2017 with two additional sampling on 31 January and 12 February 2018, representing a total of 34 sampling dates. The part of the Saint-Charles River from the dam to the DWTP is a river of order 5, with an average width of 22 m and is characterized by numerous meanders and wetlands (Brodeur et al., 2009). Raw water was collected at the DWTP before any chemical or physical treatments excepted a coarse screening at the water collection point. Water incoming at the DWTP cover a large part of the river water column (∼2 m depth) and could be considered as a representative sample of the full river water column at this point. Water was collected directly in three different sterile and nucleic acid-free 4-liter Cubitainers^TM^ (replicates) then transported at 4°C to the laboratory, and processed within 2 h. The sampling strategy and experimental procedures used in this study were detailed in the previous work focusing on the bacterial communities of the Saint-Charles River (Cruaud et al., 2019). Briefly, 400 mL of each Cubitainers^TM^ were first passed through a 20 μm mesh and then filtered through a 3 μm pore-size polycarbonate membrane filter (*large pore-size filter*) in line with a 0.22 μm Sterivex^TM^ unit (*small pore-size filter*). To evaluate replicability of the protocol, filtrations were carried out in duplicate per Cubitainers^TM^, leading in total to 204 large and 204 small pore-size filters.

The environmental metadata associated with the samples, including details on the geochemistry and climatic conditions, were previously analyzed in Cruaud et al. (2019), where further information about the measurement procedures can be found. Briefly, during the sampling period (May 2016 – February 2018), the Saint-Charles River experienced large water temperature variations from 0.7°C to 21.7°C (Figure 1 and Supplementary Table S1). Snow was on the ground from 19 November 2016 to 26 April 2017 and for the two sampling dates in January and February 2018 (turquoise arrow on Figure 1 and Supplementary Table S1). The winter period was characterized by an increase in nitrogen and nitrates/nitrites concentrations, reaching 0.88 mg N.L^–1^ and 0.58 mg N.L^–1^ on 23 February 2017, respectively (Supplementary Table S1). The sampling period was characterized by some significant rainfall events during spring, summer and autumn. Both snowmelt and rainfall events usually led to an increase in the river flow and to the opening of the floodgates to prevent flooding of the lakefront. These events also led to a decrease in alkalinity and conductivity (Supplementary Table S1).

### DNA Extraction, DNA Amplifications and Sequencing

For each sampling date, nucleic acids were extracted from two *large pore-size filters* and two *small pore-size filters* coming from two different Cubitainers^TM^ using the AllPrep DNA/RNA mini kit (QIAGEN) with modifications, as described in Cruaud et al. (2017b, 2019), for a total of 136 DNA samples. Eukaryotic 18S rRNA genes (rDNA) were amplified and sequenced following a two-step PCR library preparation as detailed in Cruaud et al. (2017a). During the first PCR step, the V4 region of the eukaryotic 18S rDNA was amplified by PCR using primers E572F and E1009R (Comeau et al., 2011). PCR conditions were the same as in Cruaud et al. (2019) with 35 cycles of amplification and a hybridization temperature of 55°C. Illumina MiSeq adaptors and barcodes were subsequently added during the second PCR step, then PCR products were pooled, purified and paired-end sequenced on an Illumina MiSeq sequencer using a V3 MiSeq sequencing kit (2 × 300 bp) at the IBIS sequencing platform (Université Laval, Canada). The raw sequencing data have been submitted to the NCBI database under BioProject accession number PRJNA486319.

### Sequencing Analyses

Sequence quality controls were performed on the raw sequence dataset with FastQC v0.11.5 (Andrews, 2012). Paired-end reads were merged using FLASH v2.2.00 (Magoč and Salzberg, 2011) with default parameters and extended maximum overlap length (300). Afterward, CUTADAPT v1.12 (Martin, 2011) was used to remove primers and filter out sequences shorter than 350 bp. Sorted sequences were then dereplicated and clustered into Operational Taxonomic Units (OTUs, 97% similarity), then putative chimeric sequences and singletons were removed using VSEARCH v.2.3.4 (Rognes et al., 2016). Finally, taxonomic assignment of the reads was performed using the *Mothur* Bayesian classifier (Schloss et al., 2009), on the Protist Ribosomal Reference database (PR2, Version 4.10.0, March 2018,Guillou et al., 2013). Analysis scripts and documentations are available in a GitHub repository^1^. OTUs affiliated with *Bacteria*, *Archaea* or *Metazoa* were removed and results were normalized so that each sample contained the same number of sequences (5,053 normalized reads per sample corresponding to the lowest number of sequences in one sample for the large-pore size filters). Since eukaryotic communities from duplicate samples strongly clustered together in dendrogram analyses with Unweighted Pair Group Method with Arithmetic mean (UPGMA) (Supplementary Figure S1), an average relative proportion of each OTU was therefore calculated for each sampling date for the subsequent analyses.

### Statistical Analyses

All statistical analyses (Bray–Curtis indexes, UPGMA, Non-metric MultiDimensional Scaling (NMDS) analyses, Non-Parametric Multivariate ANalysis Of VAriance (NP-MANOVA) Student *t*-tests and Pearson’s correlation tables) were conducted using the software environment R (v. 3.4.4) and the RStudio toolkit (v.1.0.143) implemented with *Vegan* (Oksanen et al., 2007), *stringr* (Wickham, 2015), *dendextend* (Galili, 2015), *gplots* (Warnes et al., 2015), *plotrix* (Lemon, 2006), and *corrplot* (Wei et al., 2017) packages.

SIMilarity PERcentage analysis (SIMPER) was performed using the *Vegan* package on the entire OTU dataset, including sequences obtained from large and small pore-size filters. The 25 OTUs that explained the most of the dissimilarity observed between the eukaryotic community structures of cold and warm season samples were selected for further analyses.

Interplays among these 25 OTUs and between these 25 OTUs and the bacterial OTUs detected in proportions >1% for at least one sampling date in our previous study (Cruaud et al., 2019) were investigated using Pearson’s correlation and local similarity (LS) analyses using the eLSA package (Ruan et al., 2006; Xia et al., 2011, 2016) with default parameters and no delay between sampling dates. While Pearson’s correlation coefficients detect linear relationships between OTUs during the full sampling period, the LS method might capture non-linear associations, such as predator-prey interactions for which successive positive and negative covariance may exist over the course of the time (Ruan et al., 2006; Xia et al., 2011; Carr et al., 2019). Results were represented as networks using the software environment R implemented with the *igraph* package (Csardi and Nepusz, 2006). For both Pearson’s correlation and LS networks, eukaryotic OTUs were defined as nodes and position of the nodes were determined using a same force-directed layout calculated with the Pearson’s correlations (Fruchterman-Reingold layout algorithm, using the weight parameter defined with Pearson’s correlation coefficient to increase the attraction/repulsion forces among nodes connected by higher coefficients). Connecting links (edges) represented the Pearson or LS scores with a *p*-value < 0.05. In addition, the bacterial OTUs sharing the highest Pearson and LS scores (*p*-value < 0.05) with eukaryotic OTUs (top 10 bacterial OTUs from both large and small pore-size filters) were represented around the eukaryotic networks. Correlations between two OTUs can indicate a predictive relationship that can be exploited whether or not these variables are causally related to one another. Thus, the results obtained through these analyses have to be interpreted with caution and were used in this study to search for hypothetical interactions, such as mutualism or competition, that might be experimentally tested in the future (Carr et al., 2019).

### Phylogenetic Analyses

Five phylogenetic trees (for Stramenopiles, Cryptomonads, *Spirotrichea*, *Dinophyceae*, and *Perkinsea*) were constructed to assign a finer taxonomic affiliation than obtained with the comparison against PR2 database and to infer potential nutritional capabilities for the 25 OTUs selected by SIMPER analyses (Supplementary Figures S2–S6). Sequences were compared to the GenBank, EMBL, and DDBJ databases, using the NCBI BLAST search program (Altschul et al., 1990). A total of 453 sequences composed of BLAST hits of these OTUs, sequences from PR2 database and reference publications (>1,000 bp in length) (Shalchian-Tabrizi et al., 2008; Bråte et al., 2010b; Thamm et al., 2010; Remias et al., 2013; Fernandes et al., 2016; Piwosz et al., 2016) were downloaded and aligned with MAFFT 7.310 (Katoh and Standley, 2013). Sequences of the 25 selected OTUs were aligned with this reference alignment using the “-addfragments” options in the MAFFT package (Katoh and Frith, 2012). Phylogenetic trees were estimated using maximum likelihood methods performed with RAxML HPC v8 on XSEDE (CIPRES gateway) (Miller et al., 2010; Stamatakis, 2014), using a GTR model with among-site rate variation modeled by a discrete gamma approximation with four categories. GTRCAT approximation of models was used for ML bootstrapping (1,000 replicates). The phylogenetic trees were visualized and annotated according to reference publications and PR2 taxonomic affiliations using the iTOL online tool (Letunic and Bork, 2016). Sequences of these 25 OTUs were deposited in the GenBank nucleotide sequence database under accession numbers MK618733 – MK618757. Based on their phylogenetic proximity with known lineages and considering that proximity in biological evolution could potentially lead to similar trophic mode between organisms, we tentatively classified these OTUs into three potential nutritional strategies: phototrophy, heterotrophy and mixotrophy (Supplementary Figures S2–S6 and Supplementary Table S2), with phototrophy including kleptoplasty, heterotrophy including grazing, predation and parasitism (the latter being distinguished from the heterotrophy in our figures) and mixotrophy gathering both phototrophy and heterotrophy in a same lineage. Since prediction of trophic modes based on 18S rRNA data can be challenging, only large categories of putative trophic modes were considered in this study.

## Results

### Overall Protist Diversity and Community Composition

The protist diversity was characterized based on 18S rDNA amplicon sequencing from duplicate water samples collected on large (3 μm) and small (0.22 μm) pore-size filters over 2 years from the Saint-Charles River (Figure 1). We obtained a total of 611,413 quality-filtered reads (5,053 reads per sample) that grouped within 10,500 OTUs (97% similarity), with an average of 1,217 OTUs per sampling date. Most OTUs (98.2% of the OTUs) occurred at low abundance (<1% of the reads in all samples), however, predominant OTUs (>1% of the reads in at least one sample) represented the majority of sequences (73.8% of total sequences). Only 48 OTUs (0.5% of the OTUs) were highly abundant (>5% of the reads in at least one sample) representing on average 48.9% of the reads per sample.

**FIGURE 2:**
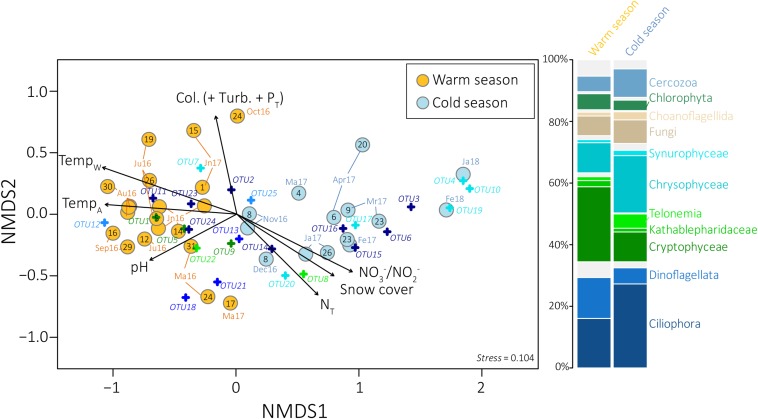
Non-metric multidimensional scaling ordination plot derived from the Bray–Curtis dissimilarity measure between samples collected on the large pore-size filters. Seasons as defined in UPGMA dendrograms are indicated in yellow and blue for the warm and the cold season, respectively. Sampling dates are indicated in black. Fitted vectors (black arrows) were added to the ordinations for the most significant variables (*envfit*, *p*-value < 0.001). The length of the arrow is proportional to the correlation between ordination and environmental variable. The 25 eukaryotic OTUs selected by SIMPER analyses were also added to the ordination (cross). The stacked barplot on the right represents the average distribution of the main microbial eukaryotic communities detected in the samples collected during the warm (left) and the cold (rigth) season as defined by UPGMA dendrograms, for the large pore-size filters. P_T_, Total phosphorus; Turb, Turbidity; Col, Apparent color; Temp_W_, Water temperature; Temp_A_, Air temperature; N_T_, Total nitrogen, Mr, March; Ma, May; Jn, Juni; Jl, July.

Bray–Curtis dissimilarity indices (Bray and Curtis, 1957, ranging from 0: the two samples have the same microbial composition, to 1: the two samples do not share any OTU) were calculated between all pairs of samples. Based on these indices, UPGMA and NMDS analyses revealed a clear seasonal pattern (Figure 2 and Supplementary Figures S7, S8). A significant difference of the eukaryotic community structure was detected between the cold and the WS (Figure 2 and Supplementary Figure S8) (NP-MANOVA, *p*-value < 0.001) with 45.8 and 48.1% of the predominant OTUs that significantly differing between the two seasons, for the large and the small pore-size filters, respectively (*t*-test, *p*-value < 0.05). Only the samples collected on 08 November showed a divergent clustering between the large and the small pore-size filters (with the cold season (CS) for the large and with the WS for the small pore-size filters, Supplementary Figure S7). Thus, the samples collected from 24 May 2016 to 24 October 2016 and from 17 May 2017 to 15 June 2017 were thereafter referred as WS samples (water temperature > 8°C, Supplementary Table S1), whereas the samples collected from 21 November 2016 to 04 May 2017 and the two sampling dates from January and February 2018 were associated to CS samples (Supplementary Figure S7, water temperature < 8°C). Bray–Curtis indices ranged from 0.98 between winter (31/01/2018) and summer (12/07/2016) samples to 0.33 between two summer samples (09/008/2016 and 16/08/2016) with an average of 0.75 over the year.

Ordination analyses of the eukaryotic community composition with environmental vector fitting suggest that water temperature and covarying factors, including air temperature, snow cover and nitrogen concentrations, as well as pH, apparent color, turbidity and phosphorus concentrations were among the main drivers of the community structure detected throughout the year (envfit, goodness of fit statistic *r*^2^ = 0.86 for water temperature, 0.67 for air temperature, 0.61 for snow cover, 0.47 for nitrogen concentrations, 0.44 for pH, 0.42 for apparent color, 0.39 for phosphorus concentrations and 0.38 for turbidity, *p*-value < 0.001, Figure 2, Supplementary Figure S8, and Supplementary Table S1).

Overall, Alveolates (mainly *Ciliophora*, *Dinoflagellata*, and *Perkinsea*), *Hacrobia* (mainly *Cryptophyceae*, *Telonemia* and *Katablepharidophyta*), Stramenopiles (mainly *Chrysophyceae* and *Synurophyceae*), *Opisthokonta* (mainly *Fungi*), *Rhizaria* (mainly *Cercozoa*), and *Archaeplastida* (mainly *Chlorophyta*) were the most predominant groups detected in our samples, both on the large and the small pore-size filters (Figure 1). Higher proportions of *Ciliophora* and *Telonemia* were detected during the CS (Figure 2 and Supplementary Figure S11) (*t*-test, *p*-value < 0.005, 27.2% in CS vs. 16.0% in WS and 4.6% in CS vs. 1.2% in WS, respectively, for the large pore-size filters, and 44.4% in CS vs. 20.9% in WS and 7.5% in CS vs. 3.8% in WS, respectively, for the small pore-size filters). *Chrysophyceae* were also detected in higher proportions during the CS but the difference was not statistically significant (Figure 2, 18.6% vs. 9.8% for the large pore-size filters and 14.2% vs. 7.5% for the small pore-size filters). In contrast, *Cryptophyta* and *Dinoflagellata* were detected in higher proportions during the WS (Figure 2 and Supplementary Figure S1, *t*-test, *p*-value < 0.005, 24.3% vs. 9.7% and 13.3% vs. 5.2%, respectively, for the large pore-size filters and 24.3% vs. 9.7% and 9.6% vs. 2.1%, respectively, for the small pore-size filters).

### Large Versus Small Pore-Size Filters

**FIGURE 3:**
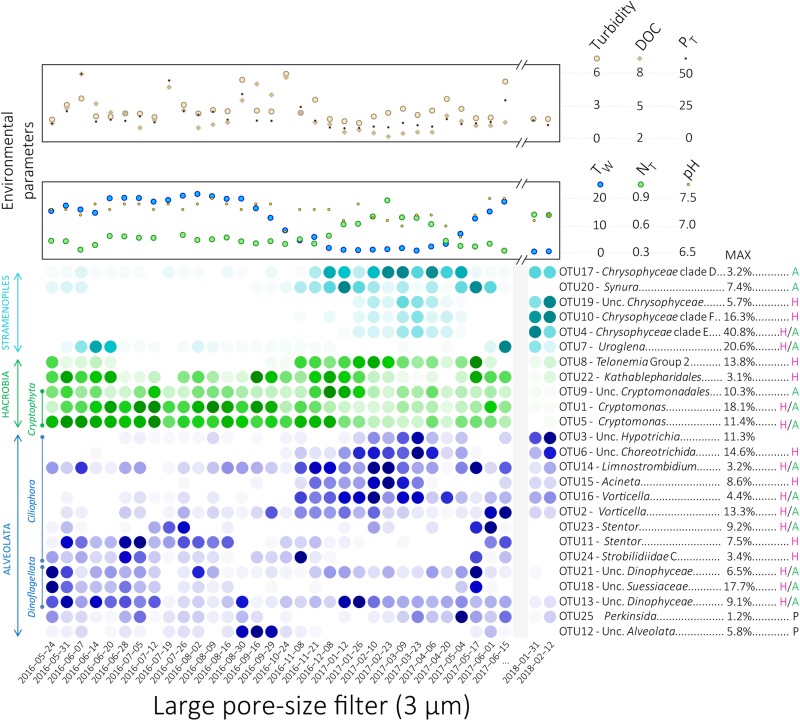
Heatmap display of the relative proportions of the 25 OTUs selected by SIMPER analyses for the large pore-size filters. The color intensity represents the relative proportion of each OTU where dark colors represent high relative proportions and light colors represent low relative proportions. The darkest colors for the highest relative proportions were defined separately for each OTU. Letters to the right of OTU names represent potential nutritional capabilities for each OTU: green “A” for potential phototrophy, purple “H” for potential heterotrophy, “H/A” for potential mixotrophy and black “P” for potential parasitism. Variations of the environmental and physico-chemical parameters highlighted using *envfit* analyses for NMDS ordination are represented at the top of the heatmap. Unc, Unclassified; P_T_, Total phosphorus, T_W_, Water temperature; N_T_, Total nitrogen.

Results obtained from the large and the small pore-size filters were quite similar (Figures 2, 3, Supplementary Figures S7–S10, and Supplementary Table S2). Many published works considered eukaryotic cells smaller than 3 μm as important component of the eukaryotic community (e.g., Medlin et al., 2006; Newbold et al., 2012; Marquardt et al., 2016). However, a large number of sequences affiliated with organisms that are well known to be bigger than the pore-size of the large pore-size filters (e.g., ciliates and dinoflagellates) was detected on the small pore-size filters (0.2–3 μm). This observation could result from potential methodological bias maybe associated with filtration failures, although different bacterial communities were observed between small and large pore-size filters (Cruaud et al., 2019). It could also result from cell plasticity and disruption. Furthermore, low PCR amplifications and insufficient sequence numbers, leading to the removal of four samples from our datasets, were observed for the small pore-size filters. For these reasons, results from the two size fractions were not averaged or pooled to avoid assignment of the same weight for the large and the small pore-size filters in our dataset. Consequently, results for the small pore-size filters are presented as Supplementary Files (Supplementary Figures S1, S7–S10), while results for the large pore-size filters are presented in the main text.

### Affiliations and Phylogenetic Placements of 25 Specific Protist OTUs

**FIGURE 4:**
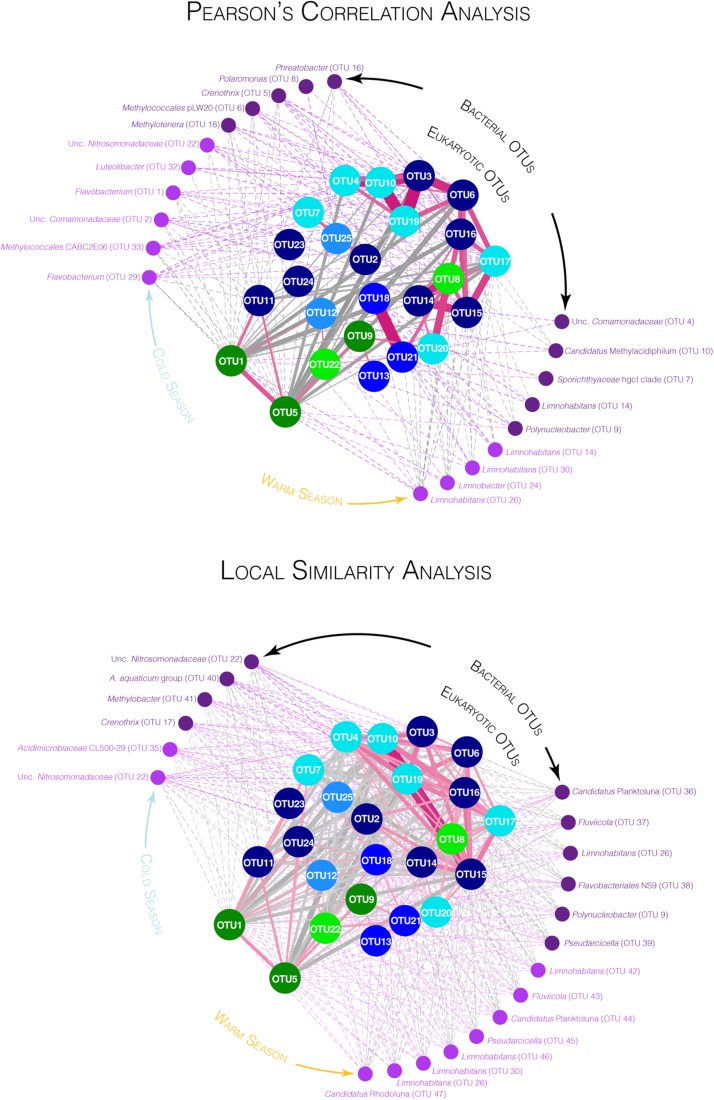
Co-varying networks for the 25 OTUs selected by SIMPER analyses for the large pore size filters with bacterial OTUs detected on both large and small fractions. Networks were constructed based on the significant Pearson’s correlation coefficients (PCC, top) and on the significant local similarity (LS) scores (bottom) between eukaryotic OTUs and between eukaryotic and bacterial OTUs (*p*-value < 0.05). Large nodes in the center of the networks represent eukaryotic OTUs (circle) with the same color code as in Figure 3. Small nodes at the periphery of the networks represent the top 10 bacterial OTUs detected on the large pore-size filters (dark purple) and the top 10 bacterial OTUs detected on the small pore-size filters (light purple) that shared the highest PCC (network on the top) or the highest LS scores (network on the bottom) with the 25 eukaryotic OTUs. Solid links (edges) represent correlation between eukaryotic OTUs and dashed links represent correlation between bacterial and eukaryotic OTUs. Pink links and gray links represent positive and negative correlations, respectively (darker and larger edges represent higher PCC or LS scores, detailed in Supplementary Table S3).

Based on SIMPER analyses, we identified 25 OTUs that explain most of the dissimilarity between seasons (39.4% of total number of sequences, cumulative contribution to the dissimilarity 38.6%). These OTUs, affiliated to Stramenopiles, *Hacrobia*, and *Alveolata*, were studied in detail (Figures 3, 4, Supplementary Figure S9, and Supplementary Table S2). Phylogenetic trees (Supplementary Figures S2–S6) were used to (i) confirm taxonomic affiliations obtained with the PR2 database, (ii) identify phylogenetically close sequences and their isolation source, and (iii) to infer potential nutritional strategies depending on their phylogenetic placements.

Six OTUs affiliated with Stramenopiles were selected (Supplementary Figure S2 and Supplementary Table S2). The OTUs 4, 7, 10, 17, and 19 were affiliated with *Chrysophyceae*. The OTU4 was close to the mixotrophic *Ochromonas tuberculata* (Bird and Kalff, 1986; Charvet et al., 2012; Stoecker and Lavrentyev, 2018) and the OTU7 was affiliated with the mixotroph *Uroglena americana* (Kimura and Ishida, 1985; Sandgren et al., 1995; Urabe et al., 1999) and formed a highly supported group with other sequences affiliated with *Uroglena* within the clade C of *Chrysophyceae*. The OTU10 was close to the colorless chrysomonads *Paraphysomonas* genus group (Caron et al., 1999; Scoble and Cavalier-Smith, 2014) and the OTU17 clustered with the *Hydrurus*-related algae (Klaveness and Lindstrøm, 2011; Remias et al., 2013) and other environmental clones isolated from high-mountain and arctic lakes in the *Hydrurus*-clade. Finally, the OTU19 formed a highly supported phylogenetic group with colorless *Spumella*-like flagellate JBC27 (Boenigk et al., 2005). Belonging to the *Synurophyceae*, the OTU20, affiliated with *Synura petersenii*, clustered with the highly supported group of the strict phototrophic *Synura* (Olefeld et al., 2018) (Supplementary Figure S2 and Supplementary Table S2).

Five OTUs affiliated with *Hacrobia* were selected by SIMPER analyses (Supplementary Figure S3 and Supplementary Table S2). Three OTUs were affiliated with *Cryptophyceae* (OTUs 1, 5, and 9), whereas one OTU was affiliated with *Telonemia* (OTU8) and another OTU with Kathablepharidales (OTU22). Two cryptophyte OTUs formed a highly supported group with the mixotrophic *Cryptomonas* (OTUs 1 and 5) (Tranvik et al., 1989; Choi et al., 2013). The third cryptophyte OTU, affiliated with unclassified *Cryptomonadales*, formed a highly supported phylogenetic group with potential phototrophic CRY2 lineage within the *Cryptophyceae* (OTU9) (Laza-Martínez et al., 2012). The OTU8 was affiliated with the heterotrophic predator lineage *Telonemia* (Klaveness et al., 2005; Bråte et al., 2010a) and the kathablepharid OTU22 formed a highly supported phylogenetic group with other uncultured freshwater eukaryotes within the kathablepharids, a group of predatory, heterotrophic biflagellates (Arndt et al., 2000) (Supplementary Figure S3 and Supplementary Table S2).

Fourteen OTUs affiliated with *Alveolata* were selected (Supplementary Figures S4–S6 and Supplementary Table S2). Nine OTUs were affiliated with *Ciliophora* (OTUs 2, 3, 6, 11, 14, 15, 16, 23, and 24), three were affiliated with *Dinoflagellata* (OTUs 13, 18, and 21), one was affiliated with *Perkinsida* (OTU25) and one OTU remained unclassified within the *Alveolata* (OTU12) according to the PR2 database. Among the *Ciliophora*, four OTUs were affiliated with *Spirotrichea* (OTUs 3, 6, 14, and 24). The OTU3 was affiliated with unclassified *Hypotrichia* and the OTU6 was affiliated with unclassified *Choreotrichida* [clustering with the highly supported group C of potential heterotrophs *Strobilidiidae* (Stoecker and Michaels, 1991)]. The OTU14, affiliated with *Limnostrombidium* within the *Strombidiida* lineage, might be able to switch from heterotrophy to phototrophy using ingested chloroplast (kleptoplasty (Stoecker and Silver, 1990; Dolan, 1992; Schoener and McManus, 2012). The last *Spirotrichea* OTU, the OTU24, grouped within the *Strobilidiidae* clade C composed of potentially heterotrophic lineages (Stoecker and Michaels, 1991) (Supplementary Figure S4 and Supplementary Table S2). Two ciliates OTUs were affiliated with *Stentor*: the OTUs 11 and 23, close to *Stentor muelleri* and *Stentor* cf. *katashimai*, which are known to contain no symbiotic green algae (Gong et al., 2007; Thamm et al., 2010) (Supplementary Table S2). Two other ciliates OTUs, the OTU2, and the OTU16, were affiliated with potentially mixotrophic *Vorticella* (feeding on bacteria but usually containing *Chlorella*) (Dolan, 1992; Jacquet et al., 2005) (Supplementary Table S2). The last ciliate OTU, the OTU15, was affiliated with the potential heterotrophic *Acineta* (Dovgal and Pesic, 2007; Gazulha and Utz, 2016) (Supplementary Table S2). Among the OTUs affiliated with *Dinoflagellata*, the OTU18 was affiliated with unclassified *Suessiaceae* close to *Biecheleriopsis adriatica* while the OTU13 and the OTU21 were phylogenetically closed and affiliated with unclassified *Dinophyceae*. These OTUs were potential mixotrophic dinoflagellates (Hansen et al., 2007; Moestrup et al., 2009) (Supplementary Figure S5 and Supplementary Table S2). The OTU25 was affiliated with *Perkinsida* within the PERK 12-17 clade among the parasitic lineage *Perkinsea* (Lepère et al., 2008; Bråte et al., 2010b; Chambouvet et al., 2014) (Supplementary Figure S6 and Supplementary Table S2). Finally, the unclassified *Alveolata* OTU12 (based on PR2 database affiliation) shared 97% sequence similarity with sequences amplified from the oligo-mesotrophic Lake Pavin and identified as perkinsid based on Chambouvet et al. (2014). This OTU was thus included in the phylogenetic tree of perkinsid that confirmed its place close to the perkinsid OTU25 within the PERK12-17 clade (Supplementary Figure S6 and Supplementary Table S2).

### Temporal Variation of the 25 Selected Protist OTUs and Correlations With Biotic and Abiotic Factors

Temporal variations of the relative proportions of the 25 protist OTUs were analyzed in detail (Figure 3 and Supplementary Figure S9). In addition, highest covariations between the relative proportions of these 25 OTUs based on Pearson’s correlation and LS analyses were explored using networks (Figure 4, Supplementary Figure S12, and Supplementary Table S3). Correlations between relative proportions of eukaryotic and bacterial OTUs [details in Cruaud et al. (2019)] were also investigated and the highest correlations were represented on the same networks (Figure 4, Supplementary Figure S13, and Supplementary Table S3). Finally, correlations have been also searched between the 25 eukaryotic OTUs and environmental parameters (Supplementary Figure S14).

The eukaryotic OTUs were distributed in the networks according to a force-directed layout, clustering together the most covariable OTUs (Figure 4). The OTUs 3, 4, 6, 8, 10, 14, 15, 16, 17, 19, and 20 were mainly detected during the CS while the other OTUs were detected throughout the year or mainly during the WS. Both the Pearson and the LS analyses showed higher covariation patterns between OTUs detected during the CS than between other OTUs. A larger number of significant correlations were detected with the LS analyses than with the Pearson’s analyses (Figure 4) but some correlations detected with the Pearson analyses were also not identified with the LS analyses (Figure 4).

The *Chrysophyceae* OTU17 as well as the three *Ciliophora* OTUs 6, 15, and 16 were detected throughout the CS and showed similar pattern of variation (Figures 2–4, Supplementary Figure S9, and Supplementary Table S3). Covariations were detected between these eukaryotic OTUs and numerous bacterial OTUs detected during the CS such as *Crenothrix*, *Methylotenera*, unclassified *Comamonadaceae*, unclassified *Nitrosomonadaceae*, *Candidatus* Nitrotoga and *Methylococcales* pLW20 (Figure 4, Supplementary Figure S13, and Supplementary Table S3). These eukaryotic OTUs were positively correlated with environmental variables increasing during the winter season such as the snow cover and the nitrogen concentrations and were negatively correlated with environmental variables decreasing during the CS such as the air and the water temperatures (Figure 2 and Supplementary Figure S14). Also mainly detected during the CS, relative proportions of the *Hypotrichia* OTU3 and the three *Chrysophyceae* OTUs 4, 10, and 19 showed similar variations reaching maximal proportions in March in winter 2017 and larger proportions during winter 2018 (up to 40.8% of the reads for the OTU4, Figures 2–4, Supplementary Figures S9, S13, and Supplementary Table S3). Covariations were detected with bacterial OTUs detected during the CS such as *Crenothrix*, *Methylococcales* CABC2E06, *Flavobacterium* or *Luteolibacter* (Figure 4, Supplementary Figure S13, and Supplementary Table S3). Although coefficients were lower, the eukaryotic OTUs 3, 4, 10, and 19 were also correlated with environmental variables that characterize the CS (Figure 2 and Supplementary Figure S14). The *Telonemia* OTU8, the *Ciliophora* OTU14 and the *Synura* OTU20 also showed the highest proportions during the CS (Figures 2, 3 and Supplementary Figure S9). However, these OTUs showed a slightly different pattern, reaching maximal proportions at the beginning of the CS as well as at the first date of the WS (17 May 2017, Figures 3, 4 and Supplementary Figures S9, S12), sharing lower correlations with the bacterial OTUs detected during the CS (Figure 4, Supplementary Figure S13, and Supplementary Table S3). These three OTUs were slightly correlated with the environmental variables associated with the CS such as the decrease in temperatures and the increase in snow cover and nitrogen concentrations (Figure 2 and Supplementary Figure S14). These three patterns (throughout the CS, higher proportions detected during the winter 2018 and beginning of the CS as well as the first date of the WS) were clearer on network build using Pearson’s correlation analyses than using LS analyses where all eukaryotic OTUs detected during the CS were connected (Figure 4).

By contrast, covariations between the eukaryotic OTUs mainly detected during the WS were less obvious, each of them showing relatively different temporal patterns. Furthermore, only few correlations were detected with the environmental variables measured in this study (Supplementary Figure S14). Some of these eukaryotic OTUs (OTUs 1, 5, 11, 12, 18, and 21) were correlated with different bacterial OTUs mainly detected during the WS, such as *Sporichthyaceae* hgcI clade and *Limnohabitans* (Figure 4, Supplementary Figure S13, and Supplementary Table S3) The OTUs affiliated with *Cryptophyceae* and Katablepharidales (OTUs 1, 5, 9, and 22) were detected throughout the year but their relative proportions remained relatively low from February to May (Figures 2–4 and Supplementary Figure S9). The *Cryptomonas* OTUs 1 and 5 were highly correlated with environmental parameters associated with the WS such as the increase in temperature and the decrease in snow cover (Figure 2 and Supplementary Figure S14). The *Uroglena* OTU7 was the only OTU affiliated with Stramenopiles showing the highest relative proportions during the WS (Figures 2, 3 and Supplementary Figure S9) but was also detected in significant proportions on 31 January 2018 (up to 12.35% for the small pore-size filters, Figure 3 and Supplementary Figure S9). The distribution of the dinoflagellates OTUs 18 and 21 reached maximal relative proportions at the end of May (Figure 3 and Supplementary Figure S9). These OTUs were highly correlated according to Pearson’s correlation analyses, showing a global covariation along the entire time of our sampling period, while only a little correlation was found with the LS analyses, revealing different local dynamics (Figure 4 and Supplementary Table S3), that preclude real relationship between these OTUs. In addition with the bacterial OTUs noted above, these two OTUs were correlated with a bacterial OTU affiliated with *Polynucleobacter* (Figure 4, Supplementary Figure S13, and Supplementary Table S3). The third dinoflagellate OTU (OTU13) fluctuated throughout the year (Figure 3 and Supplementary Figure S9). The *Ciliophora* OTUs 2, 11, 23, and 24 showed higher relative proportions during the WS. Among them, the *Stentor* OTUs 11 and 23 reached maximal relative proportions at the beginning of the WS (May and June, Figure 3 and Supplementary Figure S9) and the OTU11 was correlated with the increase in air and water temperatures (Supplementary Figure S14). The OTU24 reached maximal relative proportions on 28 June 2016 and high relative proportions on 08 November 2016 as well (Figure 3 and Supplementary Figure S9). The *Perkinsida* OTU25 was detected intermittently throughout the year and mainly on the small pore-size filters with highest proportions on May 2017 (up to 12.6% on these filters, Figure 3, Supplementary Figure S9, and Supplementary Table S2). This OTU was highly correlated with a bacterial OTU affiliated with *Polaromonas* (Figure 4, Supplementary Figure S13, and Supplementary Table S3). This OTU was also correlated with the increase in river flow, the opening of the dam floodgates and the amount of rainfall 3 days before sampling (Supplementary Figure S14). Finally, the OTU12 was mainly detected on 3 sampling dates, in late summer 2016 (up to 19.8% for the small pore-size filters on 29 September, Figure 3, Supplementary Figure S9, and Supplementary Table S2) and was highly correlated with two bacterial OTUs mainly detected during the WS and affiliated with *Sporichthyaceae* hgcI clade and *Candidatus* Methylacidiphilum (Figure 4, Supplementary Figure S13, and Supplementary Table S3) and with the increase of the dissolved organic carbon concentration in the water (Supplementary Figure S14).

## Discussion

### Marked Seasonality of Overall Composition of Protist Communities

Using 18S rDNA sequencing, a high diversity of small eukaryotes was observed in the Saint-Charles River with members of all eukaryotic superphyla (Figure 1) and numerous rare OTUs. The eukaryotic microbial communities were dominated by *Alveolata*, *Hacrobia*, and Stramenopiles while *Opisthokonta*, *Rhizaria*, and *Chlorophyta* were detected in smaller proportions (Figure 1). If this result is consistent with many reports from freshwater ecosystems (Lefranc et al., 2005; Richards et al., 2005; Šlapeta et al., 2005; Triadó-Margarit and Casamayor, 2012; Taib et al., 2013; Simon et al., 2015), the large variation in 18S rRNA gene copy numbers among eukaryotic species and between growth phases [1 to highest estimates around 37,000 copies per cell in diatoms and 310,000 copies in ciliates (Prokopowich et al., 2003; Godhe et al., 2008; Gong et al., 2013)] might have led to overestimation of the relative abundance of some lineages compared to others, as previously mentioned (e.g., Mangot et al., 2009; Gong et al., 2013; Ren et al., 2018). We thus avoided as far as possible to compare relative proportions between the different lineages and focused on the dynamics of eukaryotic OTUs over months and seasons.

Eukaryotic communities showed a clear seasonal pattern with different communities between winter and summer (Figure 2 and Supplementary Figures S7, S8), as a probable consequence of the contrasted environmental conditions. This pattern was repeated during the 2nd year of sampling, suggesting an annual eukaryotic community cycle, which is consistent with previous investigations on lakes (Nolte et al., 2010; Bock et al., 2014), ponds and brooks (Simon et al., 2015). This seasonal pattern was correlated with summer and winter-associated contrasting parameters such as air and water temperatures, snow cover and nitrogen concentrations and has also been observed for the bacterial communities of the Saint-Charles River (Cruaud et al., 2019). This suggests that both bacterial and eukaryotic communities undergo deep modifications of their community composition, reflecting the dramatically different seasonal and environmental conditions of the region (Figure 2 and Supplementary Table S1) and confirming the structuring role of environmental selection in this ecosystem.

*Ciliophora*, *Chrysophyceae*, and *Telonemia* were detected in higher proportions during the CS, whereas *Cryptophyta* and *Dinoflagellata* were detected in higher proportions during the WS (Figure 2 and Supplementary Figure S11). Although DNA might not directly indicate activity or viability of the cells and the fact that 18S rDNA relative proportions could not be considered as abundances due to different copy numbers and primer efficiency, the successive increases and decreases in relative proportions of specific eukaryotic lineages during the CS (Supplementary Figure S10) suggest that, despite the ice-cover and the very low water temperature during this season (Figure 1 and Supplementary Table S1), at least a part of the eukaryotic microbial community could potentially be active and growing in the river and/or in the lake feeding the river. Furthermore, phylogenetic trees indicated that some 18S rRNA genes sequenced from winter samples clustered with sequences recovered from cold environments (ice, moutain lakes) (Klaveness and Lindstrøm, 2011; Charvet et al., 2012; Remias et al., 2013), supporting the viability of these lineages during the CS. This may reveal a potential better tolerance to cold conditions or different strategies for overwintering among these lineages. This change in microbial community composition was also observed for the bacterial community, with increasing proportion of potential methane and nitrogen cycling bacteria (e.g., bacterial OTUs 5, 6, 17, 18, 22, 33 and 41 in Figure 4) in winter, which was consistent with environmental conditions during this season. Together these results suggest that the winter season is probably not a dormant season for prokaryotic and eukaryotic microorganisms. However, we cannot exclude that a non-negligible part of the observed diversity may overwinter in a dormant or resting state (Sommer et al., 2012).

While *Ciliophora* are mainly considered as predators of bacteria (Lefranc et al., 2005; Richards and Bass, 2005) and *Cryptophyta* are assumed to be mainly photoautotrophic (Lepère et al., 2008; Grujcic et al., 2018), plastid-retention and true symbiosis with algal cell in *Ciliophora* as well as bacterivory in *Cryptophyta* have been also reported (Dolan, 1992; Stoecker et al., 2017; Grujcic et al., 2018; Stoecker and Lavrentyev, 2018), challenging the attribution of a consensus nutritional mode for these large groups. Therefore, we selected and analyzed in detail 25 predominant OTUs that fluctuated throughout the sampling period to investigate the freshwater microeukaryote ecology and the potential variations of nutritional mode throughout the annual cycle (Figure 3 and Supplementary Figure S3).

### Life in a River Covered by Ice

The CS was characterized by a broadly constant low water temperature and ice- and snow-cover on the river and on the upstream lake (Figure 1 and Supplementary Table S1). These conditions are usually associated with limited atmospheric gas exchanges and reduced particulate inputs in the water (Catalan, 1992; Bertilsson et al., 2013; Hampton et al., 2017), as well as reduced macro- and mega-faunal predatory pressure, although zooplankton and fish predation may be high even in winter (Jeppesen et al., 2004; Sørensen et al., 2011; Sommer et al., 2012). Taxonomic comparison with known representatives of the selected OTUs that predominated during winter revealed that the potential for phototrophy and phagotrophy could occur in the river during the CS (Figure 3, Supplementary Figure S3, and Supplementary Table S2). Inhibition of photosynthesis at low temperatures has been previously reported (Murata, 1989; Anesi et al., 2016) but specific physiological adaptations have been proposed for lineages in cold ecosystems, such as the *Hydrurus-*clade that includes the OTU17 (Supplementary Figure S5 and Supplementary Table S2) (Klaveness and Lindstrøm, 2011; Remias et al., 2013), suggesting that eukaryotic primary productivity might be maintained under ice. During the CS, the bacterial communities also changed in the river (Cruaud et al., 2019), suggesting a modification of the potential prey availability. Eight predominant eukaryotic OTUs with heterotrophic or mixotrophic potential were positively correlated with various winter-associated bacterial lineages, such as *Crenothrix*, *Candidatus* Nitrotoga and *Methylococcales* pLW20 (Cruaud et al., 2019; Figure 4, Supplementary Figure S13, and Supplementary Table S3). While these correlations might indicate that the same or related environmental factors (e.g., temperature, nitrogen, Figure 2, Supplementary Figure S14, and Supplementary Table S1) drive bacterial and eukaryotic populations, it might also suggest potential variation in prey availability, supporting an influence of the bacterial community composition on heterotrophic eukaryotes and *vice versa* (Jezbera et al., 2005, 2006; Pernthaler, 2005; Massana et al., 2009; Šimek et al., 2013, 2018; Grujcic et al., 2018). Together these results suggest that winter conditions did not preclude the use of light for photosynthesis or the existence of a potential grazing pressure on bacterial communities and that important mechanisms could occur during this season as emphasized in the re-evaluation of the Plankton Group Ecology (PEG) model of seasonal succession of phytoplankton and zooplankton biomasses in lake ecosystems (Sommer et al., 2012). However, these conditions may select a different fraction of the eukaryotic community that is more adapted and competitive under winter conditions than lineages identified in summer, as previously reported (Brown et al., 2004; Gyllström et al., 2005).

### Winter Comes Every Year but Each Winter Is Different

Although the overall community structure remained relatively similar during the two sampled winters (Figure 2 and Supplementary Figures S7, S8), a group of covarying OTUs that were mainly detected in March during the winter 2017 showed very high proportions during the winter 2018 (OTU3 within the *Ciliophora* and OTUs 4, 10, and 19 within the *Stramenopiles*, Figures 3, 4, and Supplementary Figure S9, and Supplementary Table S2), highlighting potential changes in environmental conditions between winters. The winter 2018 was characterized by a particularly important mid-winter thaw in January (maximal air temperature 9.3°C on 12 January 2018, Figure 1), that led to an unusual increase in river flow and a massive flooding of the watershed. Such events could potentially result in nutrient inputs from the watershed. Furthermore, below freezing temperatures in the following days coupled with the residence time of the water in the lake [32 days in average (APEL, 2015)] could have allowed the persistence of the disturbance effect over a long period of time. Thus the difference observed in eukaryotic microbial community composition between the two winters might be explained by this mid-winter weather perturbation. Interestingly, while only a limited part of the protist community was impacted, the eukaryotic microbial community composition was more altered than the bacterial communities (Cruaud et al., 2019), suggesting differences in the response time or sensitivity between the eukaryotic and the bacterial communities to environmental changes. Based on their taxonomic proximity with known species, heterotrophic and mixotrophic lifestyles were inferred for the eukaryotic OTUs detected in high proportions during the winter 2018. Therefore, they might have been triggered directly by nutrient inputs from the watershed, boosted by potential increase of light penetration in the water, or indirectly activated through the predation of specific organic matter degrading bacteria, such as *Polynucleobacter* that was also detected in unusually high proportions during the winter 2018 (affiliated as *Polynucleobacter asymbioticus* on Silva release 128 Cruaud et al., 2019). This *Polynucleobacter* lineage, that was mainly detected in strong proportion during the WS, is known to exploit photodegradation products of humic substances (Hahn et al., 2012) and to be consumed by a wide range of bacterivorous eukaryotes (Grujcic et al., 2018). However, the emergence of *Polynucleobacter* in the winter 2018 was not associated with a simultaneous growth of summer bacterivorous eukaryotes, suggesting that environmental conditions (e.g., cold temperature) apply a stronger selection on bacterivorous eukaryotic lineages than the availability of bacterial prey.

### Complex Temporal Dynamics During the Ice-Free Period

The relative proportions of the OTUs detected during the WS were more fluctuant than during the CS and only few correlations were detected among them (Figure 4, and Supplementary Figure S12, and Supplementary Table S3), suggesting a more complex temporal dynamic of the eukaryotic microbial community during this season. This results might be directly due to larger variations in environmental conditions (e.g., frequent rainfall events and runoff, water temperature fluctuating from 8°C to 23°C, changes in the watershed input such as pollen, leaf fall or erosion, Figure 1 and Supplementary Table S1) that might provide a large variety of niche opportunities for eukaryotic microorganisms. The environmental conditions during the WS could also impact the potential bacterial prey community composition (Cruaud et al., 2019) and also change the predation by higher trophic levels (Sommer et al., 1986, 2012).

At the beginning of the WS, different OTUs, related to major identified groups (*Ciliophora*, *Hacrobia*, and *Stramenopiles*) were detected in higher proportions (OTUs 8, 14, 18, 20, 21, 22, 23, and 24 Figure 3 and Supplementary Figure S9). While the *Telonemia* OTU8, the *Limnostrombidium* OTU14, the *Synura* OTU20 and the *Dinophyceae* OTU21 were detected throughout the CS and the *Suessiaceae* OTU18, the *Stentor* OTU23 and the *Strobilidiidae* OTU24 were rather detected in very low proportions during this season (CS), all these OTUs were characterized by a sudden increase in relative proportions on 17 May 2017 (Figures 3, 4 and Supplementary Figure S9). This period could correspond to the spring bloom as described in the PEG model (Sommer et al., 1986, 2012). After the spring break up in mid-April, the Saint-Charles Lake, the main source of the Saint-Charles River, was totally ice-free in early May. At this period, water of the lake mix once the water temperature reach 4°C. The mixing of the lake, as well as the large water inputs produced by snow- and ice-melting in spring, likely bring fresh nutrients which could intermittently fuel the eukaryotic community and enrich it with additional species from snow, ice, deeper waters of the lake or soil leaching. Thus, toward the end of winter, increasing water temperature, light and nutrient availability could allow the growth of fast-growing protists that overwintered in an active or a resting stage with early termination of the diapause phase (Sommer et al., 1986, 2012), as potentially illustrated by the OTUs described above and supporting that the growing season does not start from zero after the winter season (Sommer et al., 2012). Moreover, some of these OTUs were also detected in higher proportions at the beginning of the CS (OTUs 8, 14, 20, and 24). This period correspond to the fall turnover in the Saint-Charles Lake (Wetzel, 2001; Boulé et al., 2010), leading to the redistribution throughout the water column of the nutrients isolated in the hypolymnion during the summer season and potentially modifying the microbial community composition through nutrients or bacteria intake (Cruaud et al., 2019). By contrast with the WS, the beginning of the CS is not characterized by an increase in light supply and in water temperature, suggesting that these lineages might rather respond to an increase in nutrient availability or another confounding factor rather than an increase in light supply, as suggested as main starter for the spring bloom in the PEG model (Sommer et al., 1986, 2012).

After this early summer stage, we observed an increase in relative proportions of different OTUs, such as the *Cryptomonas* OTUs 1 and 5 and the *Stentor* OTU11 (Figure 3 and Supplementary Figure S9). These OTUs were correlated with the increase in air and water temperatures (Supplementary Figure S14) and with the relative proportion of some bacterial lineages mainly detected during the WS, such as *Limnohabitans*, *Limnobacter* or *Sporichthyaceae* hgcI (Figure 4, Supplementary Figure S13, and Supplementary Table S3), which are known prey for bacterivorous eukaryotes (Šimek et al., 2013, 2018; Grujcic et al., 2018; Piwosz et al., 2018). These results suggest that these potentially mixotrophic or heterotrophic OTUs could be more competitive in the middle of summer and could be slower growing species than the OTUs detected in early summer as suggested in the PEG model (Sommer et al., 1986). In addition, other eukaryotic OTUs were detected more sporadically during the WS (*Uroglena* OTU7, Unc. *Alveolata* OTU12, and *Stentor* OTU23) or both during the warm and the CS (Unc. *Dinophyceae* OTU13) with few correlations with the main environmental parameters (Supplementary Figure S14). Punctual events, such as a transient increase in phosphorus (07 June 2016, 15 June 2017) or nitrogen concentrations (CS), could explain these kinds of temporal dynamics. Likewise, the OTU12 and the OTU25, affiliated with the parasitic lineage *Perkinsidea* (Lepère et al., 2008; Chambouvet et al., 2014) were also detected sporadically throughout the sampling period. Moreover, the *Perkinsida* OTU25 was strongly correlated with a bacterial OTU affiliated with *Polaromonas* (Figure 4, Supplementary Figure S13, and Supplementary Table S3), highlighting potential hosts for this *Perkinsidae* OTU. The detection of these parasitic lineages among the main protist lineages suggests an important role of parasitism in regulation of population dynamics in freshwater ecosystems, as previously suggested (Lefranc et al., 2005; Lepère et al., 2008; Sommer et al., 2012; Mangot et al., 2013).

Together, these results might indicate potential differences among protist lineages detected during the WS such as in generation times, resistance to grazing, susceptibility to infection by virulent parasites or sensitivity to nutrient and prey availability. According to the PEG model, the plankton community during the ice-free period follows successive stages starting from a phytoplankton spring bloom followed by the development of zooplankton in response to food availability. This growth of zooplankton leads to the decline of early summer lineages, which is known as the “clear water phase” (Sommer et al., 1986, 2012). Subsequently, food limitation and fish predation limit the zooplankton biomass allowing the increase of zooplankton preys (Sommer et al., 1986, 2012). Interestingly, the variations observed in our study in the protist community composition and diversity throughout the WS did not reveal clearly separated groups for these successive stages. Without any protist, zooplankton and other predators (e.g., fish) community quantifications, the stages of plankton biomass succession cannot be clearly distinguished in our dataset. However, our study suggests that beyond the total biomass changes, complex and numerous species replacements also occur in freshwater ecosystems during the WS.

## Conclusion

Our study shows a pronounced seasonal clustering of eukaryotic microbial communities confirming the important role of environmental selection in freshwater ecosystems and the necessity of temporal series to comprehensively assess the biodiversity of these systems. Although all nutritional modes (autotrophy, heterotrophy, mixotrophy, and parasitism) were potentially identified throughout the year, complex patterns of distribution have been observed suggesting a large complexity of factors and interactions that could shape the temporal dynamics of the microbial eukaryotic community. While the modulation of grazing, photosynthesis and growth rates by water temperature could be an important variable controlling the annual dynamics of eukaryotic microbial populations, our results suggest that other factors such as the availability of specific bacterial and eukaryotic prey, variations in nutrients concentrations and the presence of various predators could also greatly influence the eukaryotic community composition warranting further investigations. Thus, understanding the underlying causes of eukaryotic microbial community temporal dynamics is highly complex and will require considerable efforts and an adequate way to understand this specific part of microbial ecology. However, such efforts are crucial considering the important impact of microbial eukaryotes on the global functioning of freshwater ecosystems.

## Data Availability Statement

The datasets generated for this study can be found in NCBI, PRJNA486319, MK618733 – MK618757.

## Author Contributions

PC, CD, AC, MR, and SC designed the study. PC and M-SF collected the samples. PC performed the laboratory work, sequencing data analyses, and statistical analyses. PC, AV, and SC analyzed and interpreted the results. PC and AV wrote the manuscript with revisions by SC, MR, CD, and AC.

## Conflict of Interest

The authors declare that the research was conducted in the absence of any commercial or financial relationships that could be construed as a potential conflict of interest.

## Footnotes

1 https://github.com/CruaudPe/MiSeq_Multigenique
