# Supplementary figures and images for: Annual Protist Community Dynamics in a Freshwater Ecosystem Undergoing Contrasted Climatic Conditions: The Saint-Charles River (Canada)
Source: Front Microbiol. 2019 Oct 16;10:2359. doi: 10.3389/fmicb.2019.02359 (PMC6805768; doi:10.3389/fmicb.2019.02359)

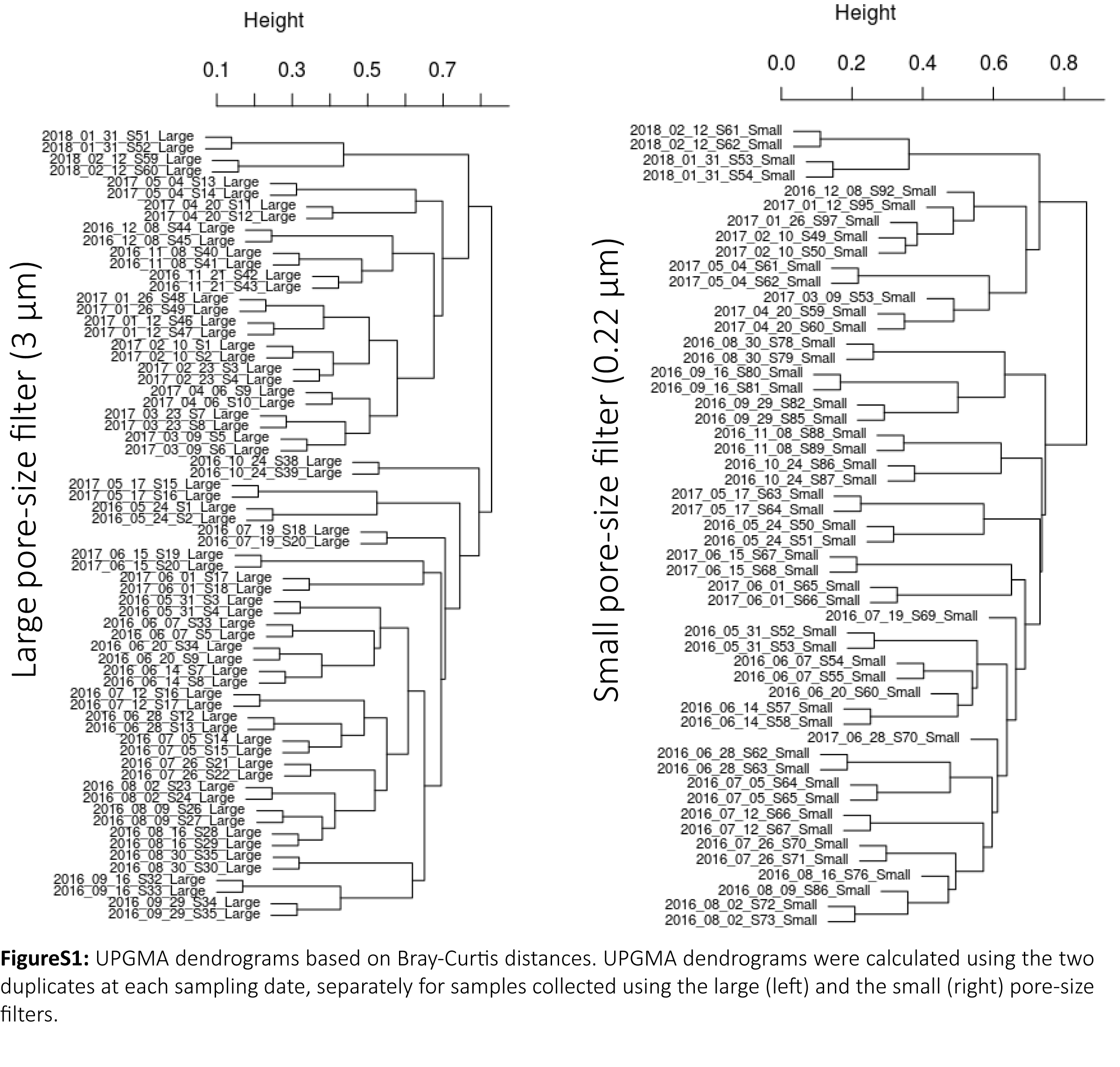

Supplement: Supplementary file 1 [file Image_1.TIF]

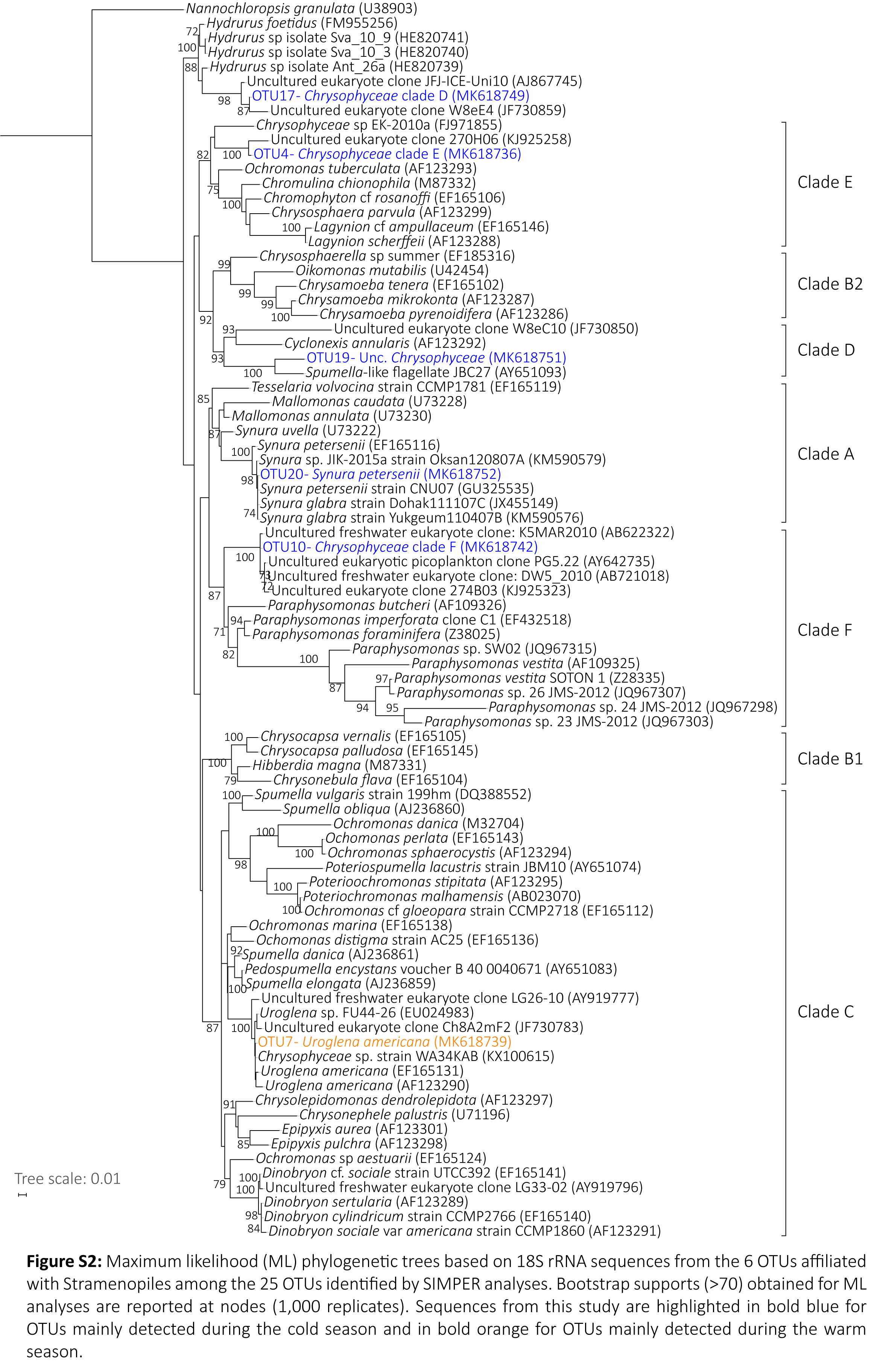

Supplement: Supplementary file 2 [file Image_2.TIF]

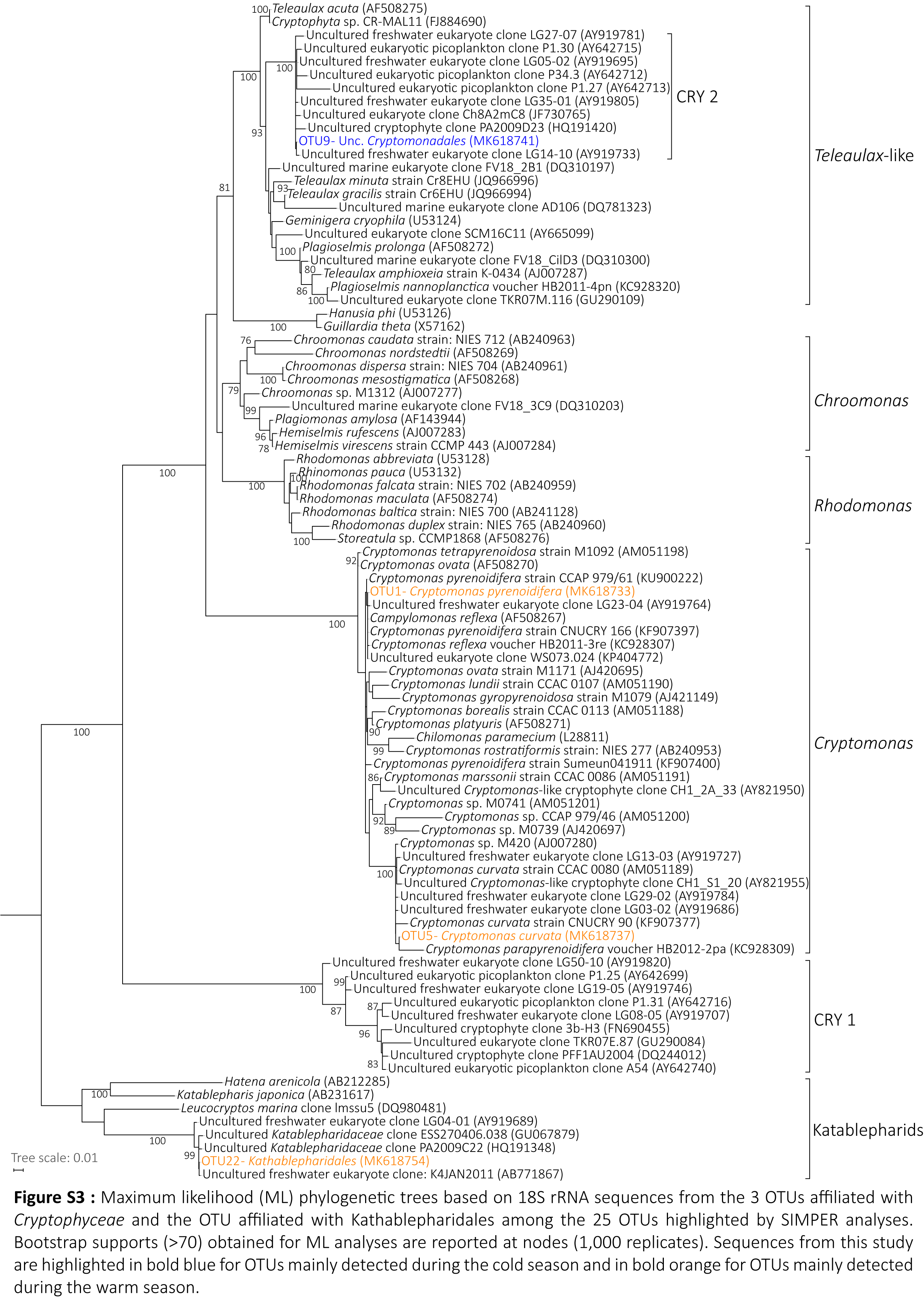

Supplement: Supplementary file 3 [file Image_3.TIF]

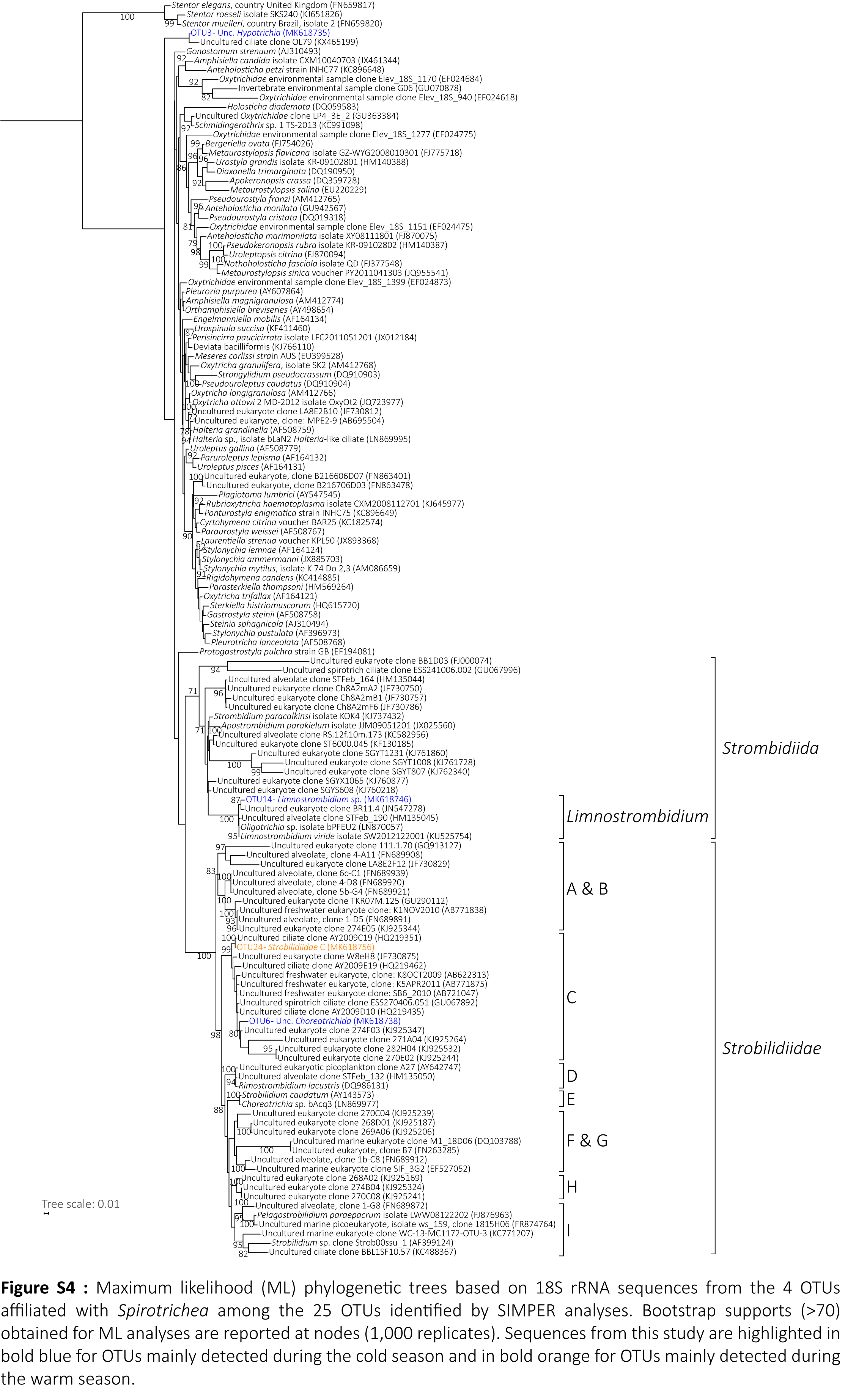

Supplement: Supplementary file 4 [file Image_4.TIF]

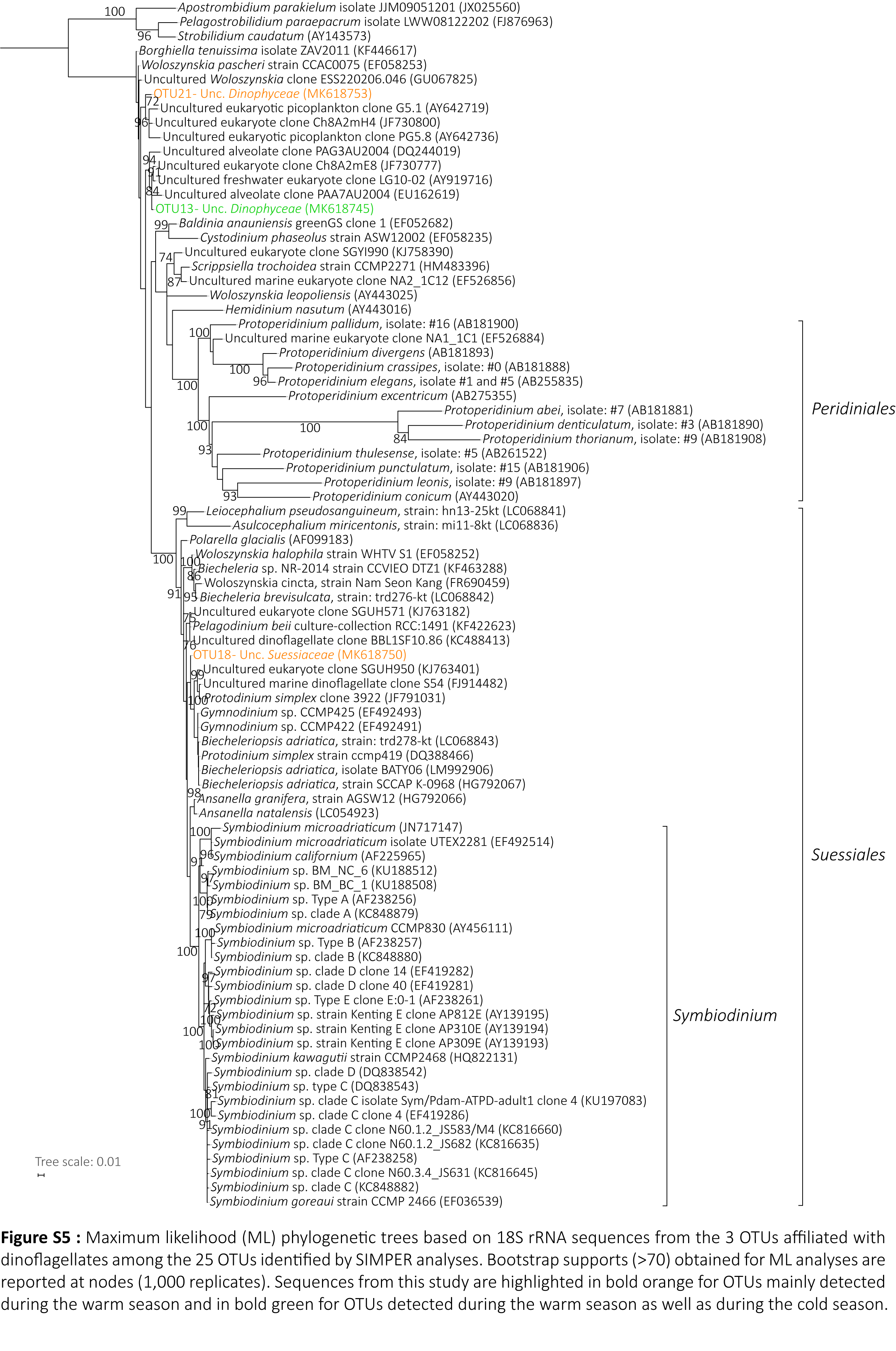

Supplement: Supplementary file 5 [file Image_5.TIF]

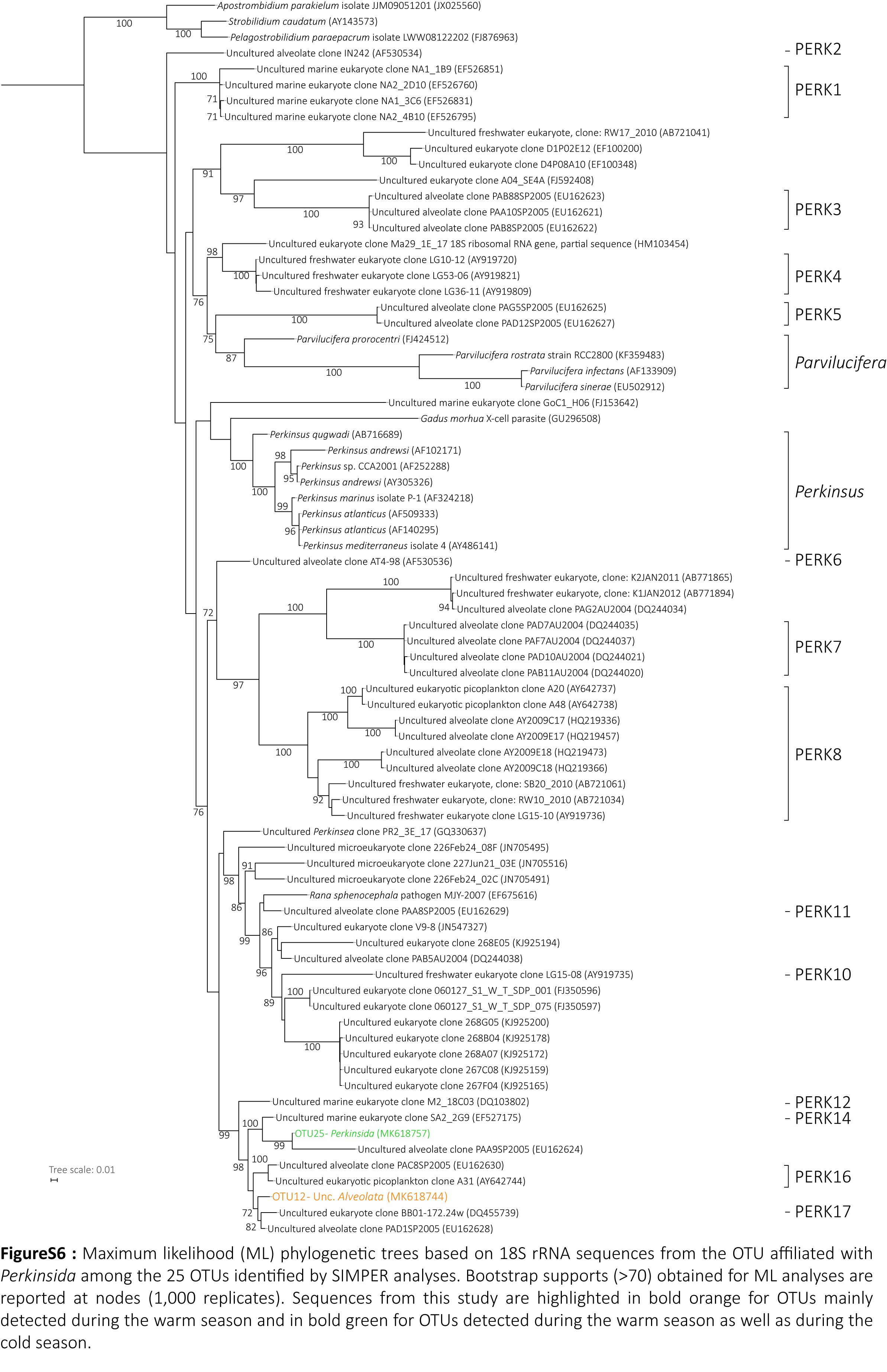

Supplement: Supplementary file 6 [file Image_6.TIF]

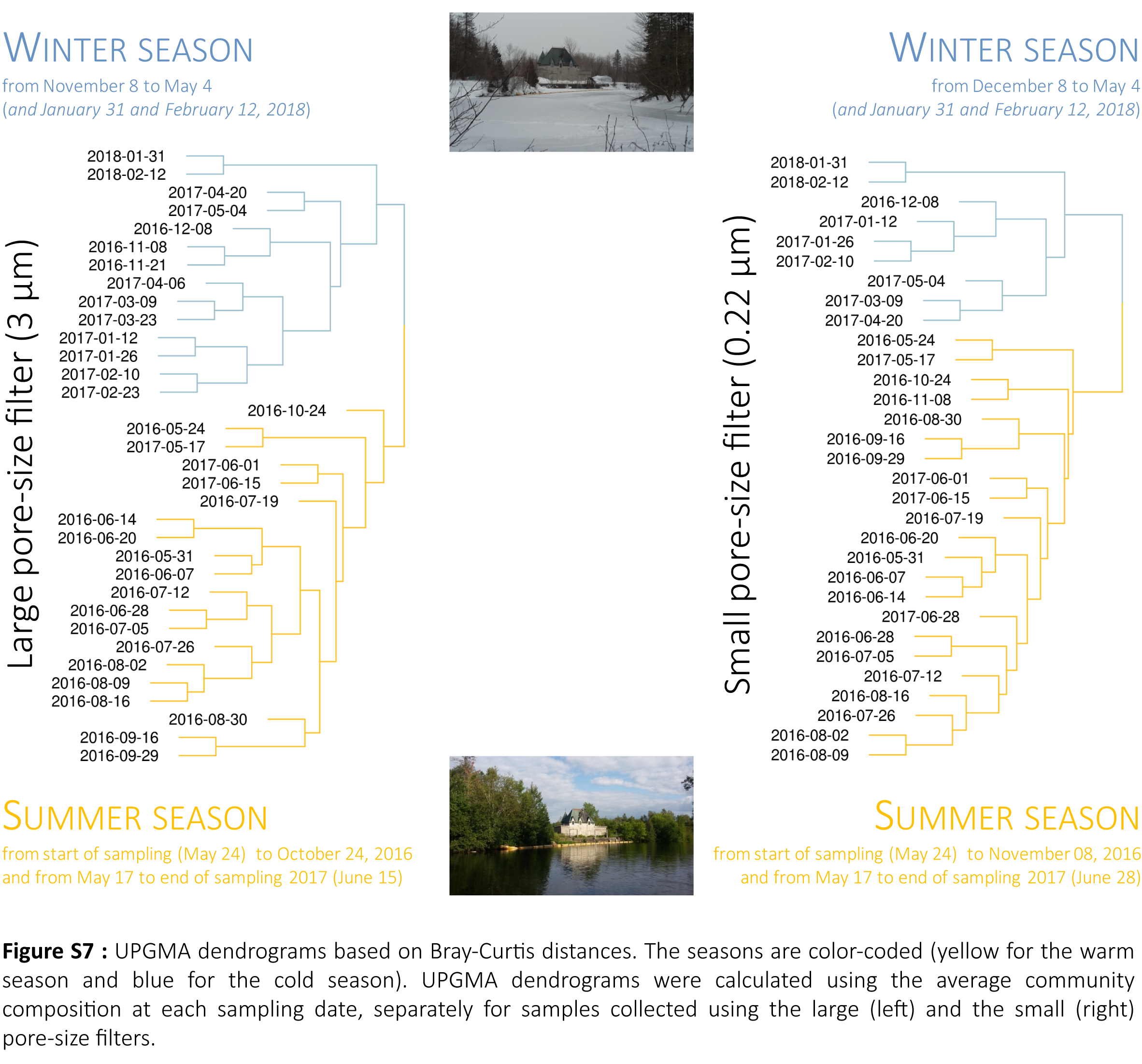

Supplement: Supplementary file 7 [file Image_7.TIF]

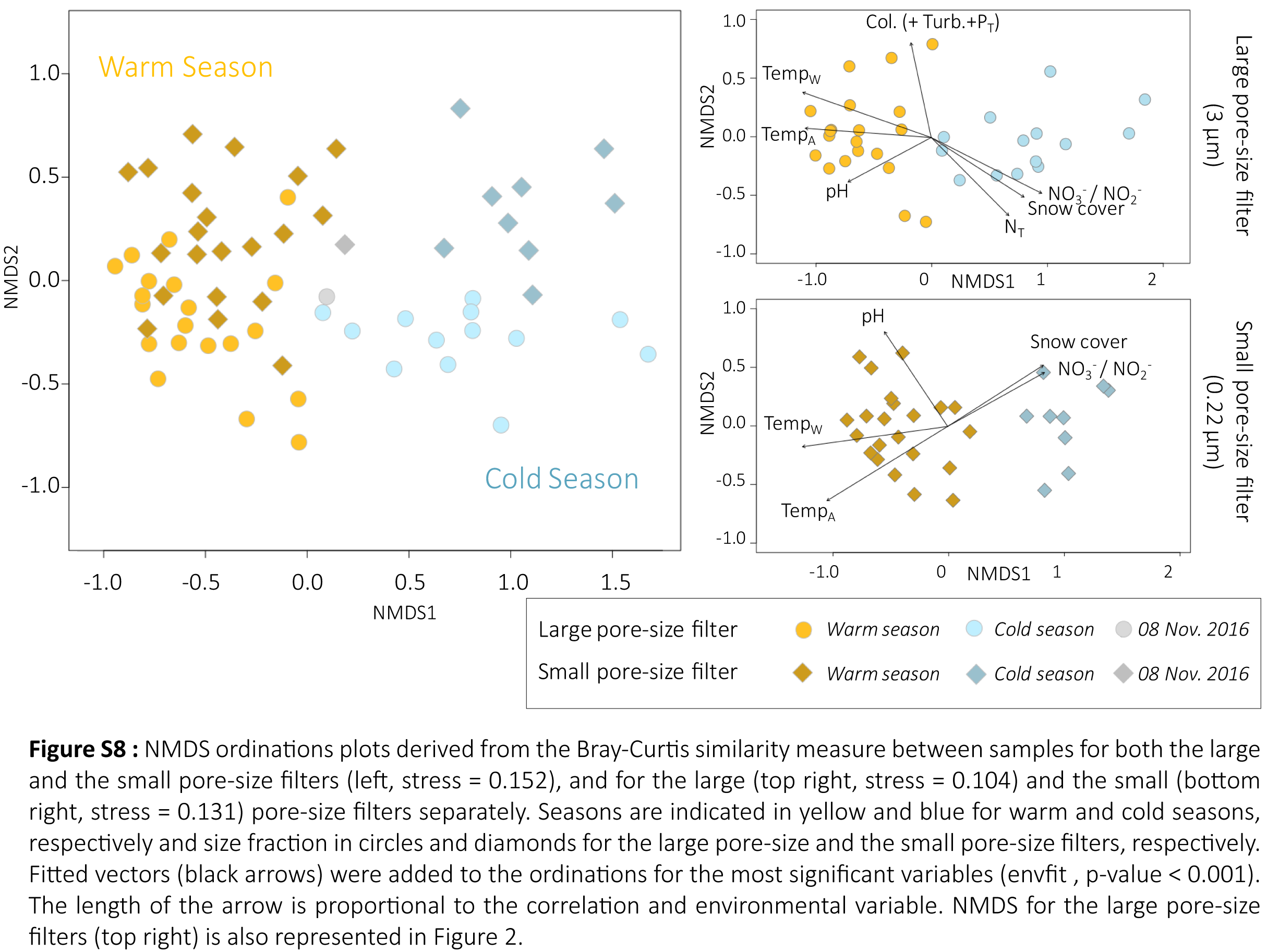

Supplement: Supplementary file 8 [file Image_8.TIF]

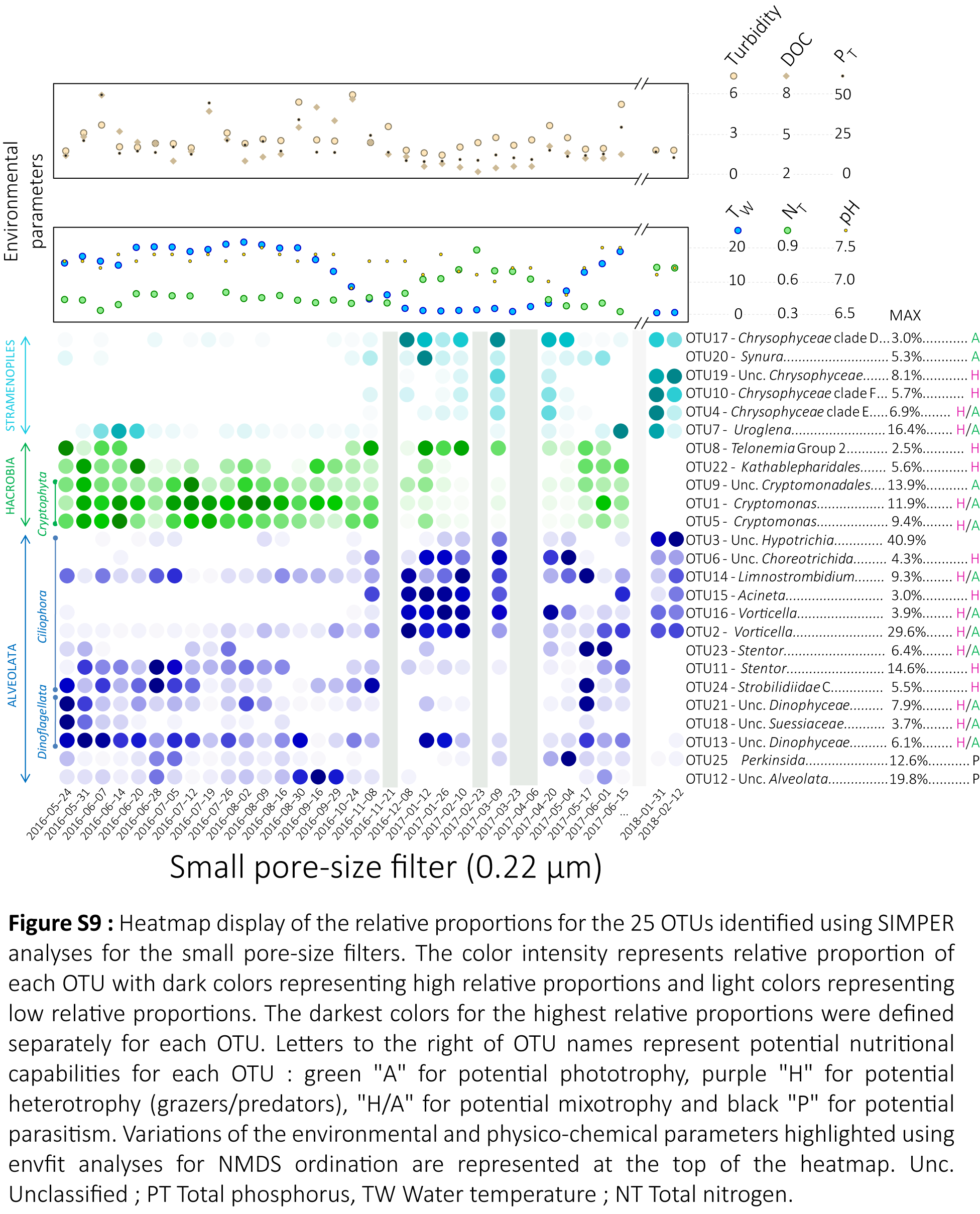

Supplement: Supplementary file 9 [file Image_9.TIF]

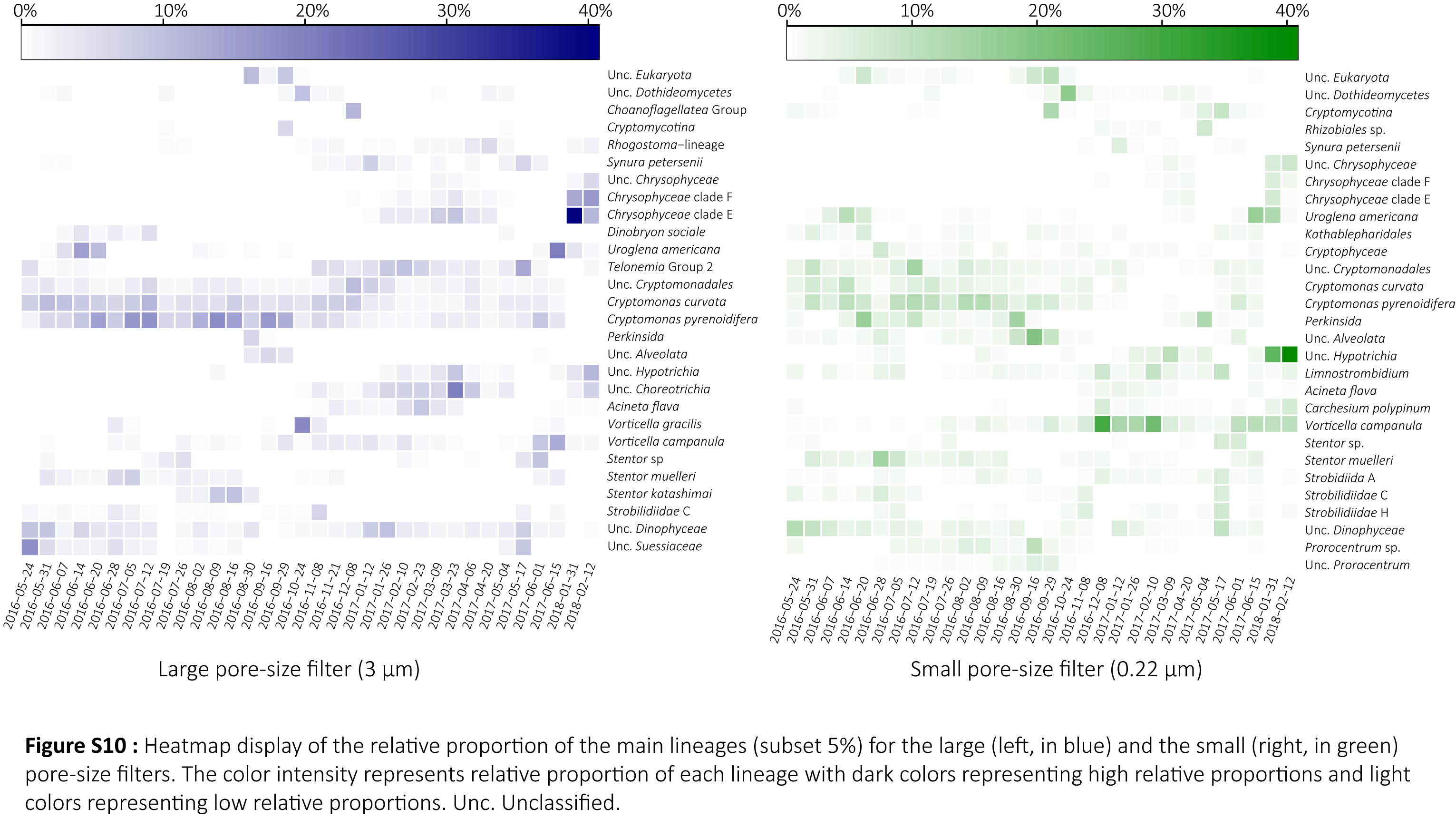

Supplement: Supplementary file 10 [file Image_10.TIF]

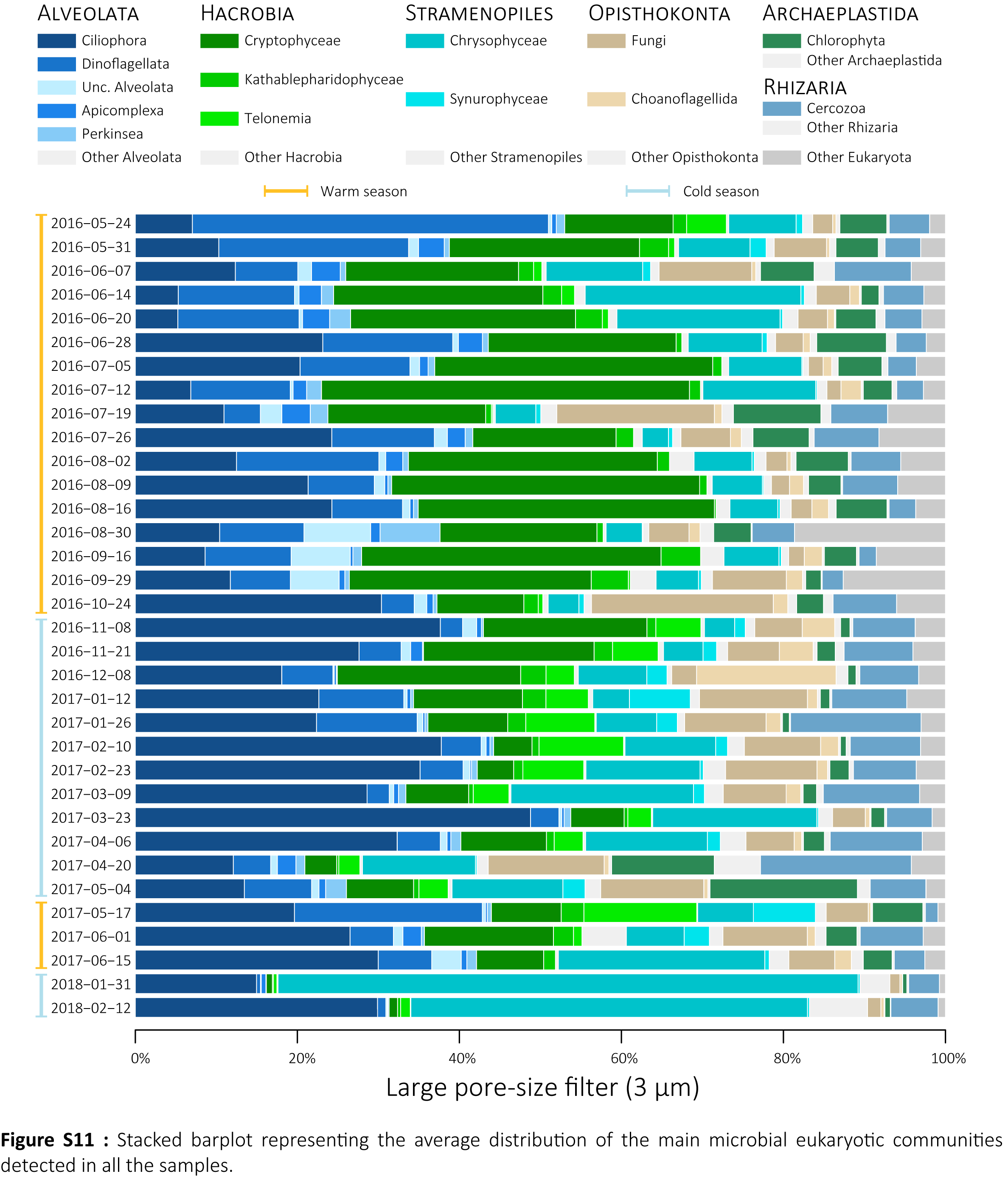

Supplement: Supplementary file 11 [file Image_11.TIF]

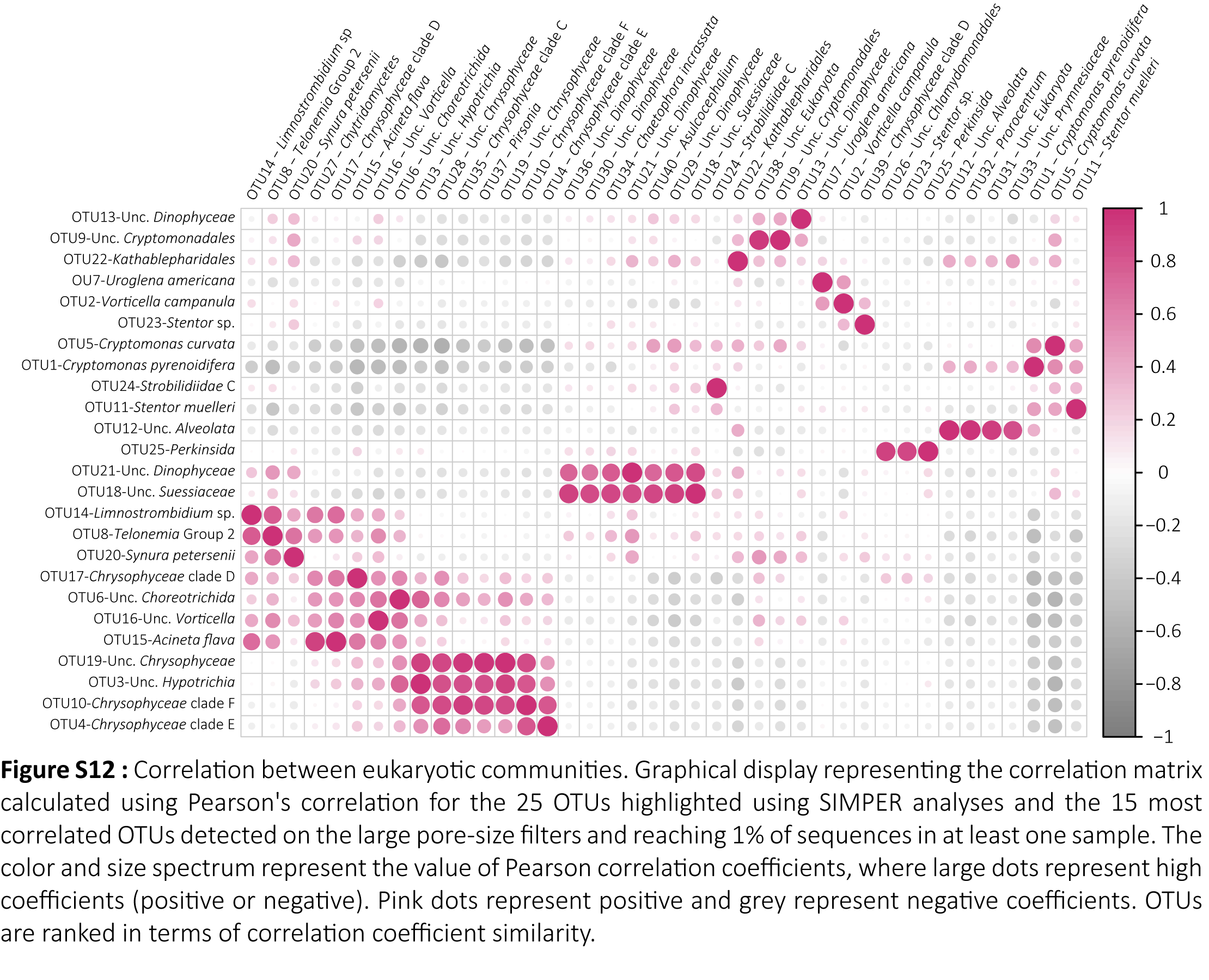

Supplement: Supplementary file 12 [file Image_12.TIF]

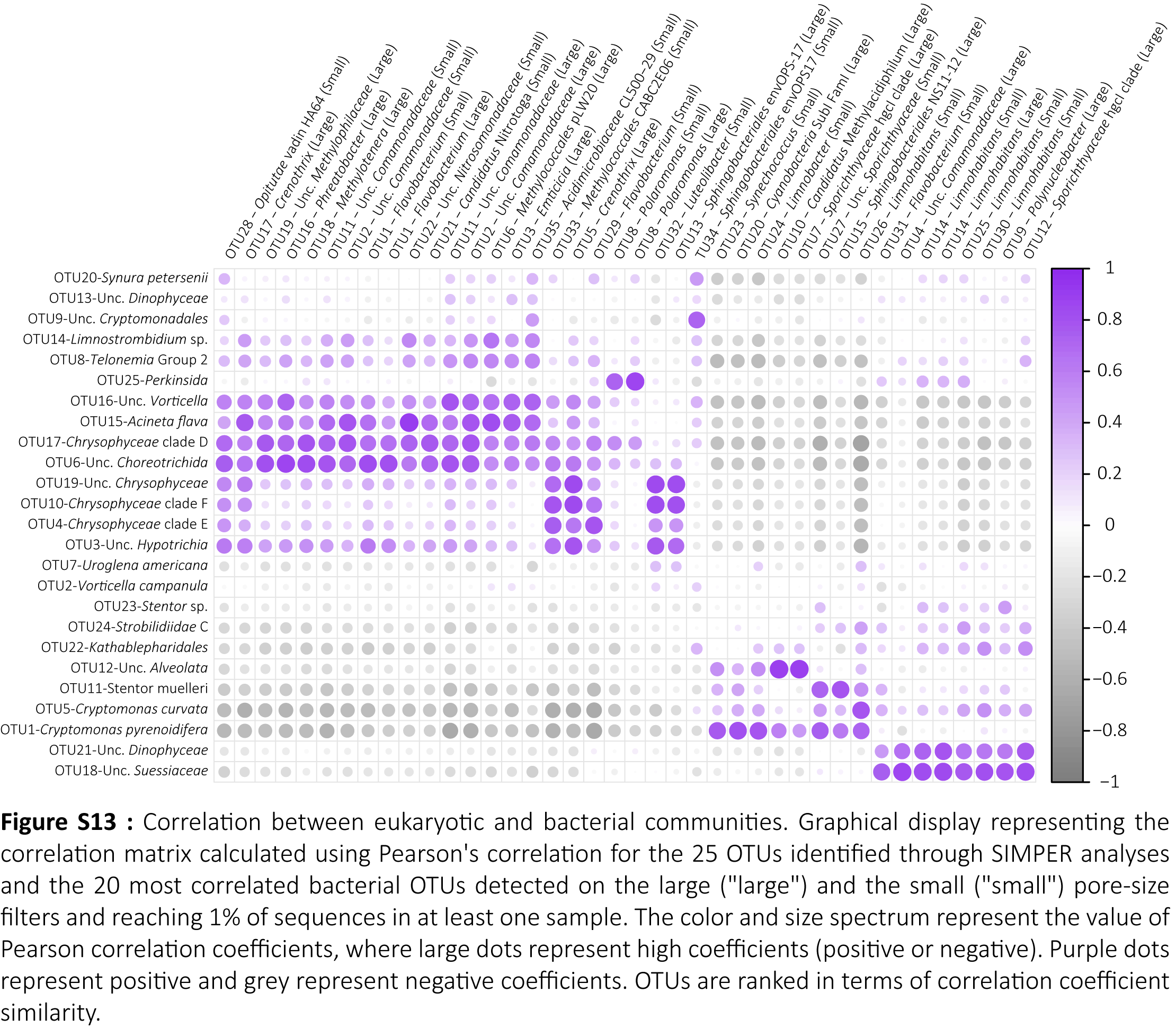

Supplement: Supplementary file 13 [file Image_13.TIF]

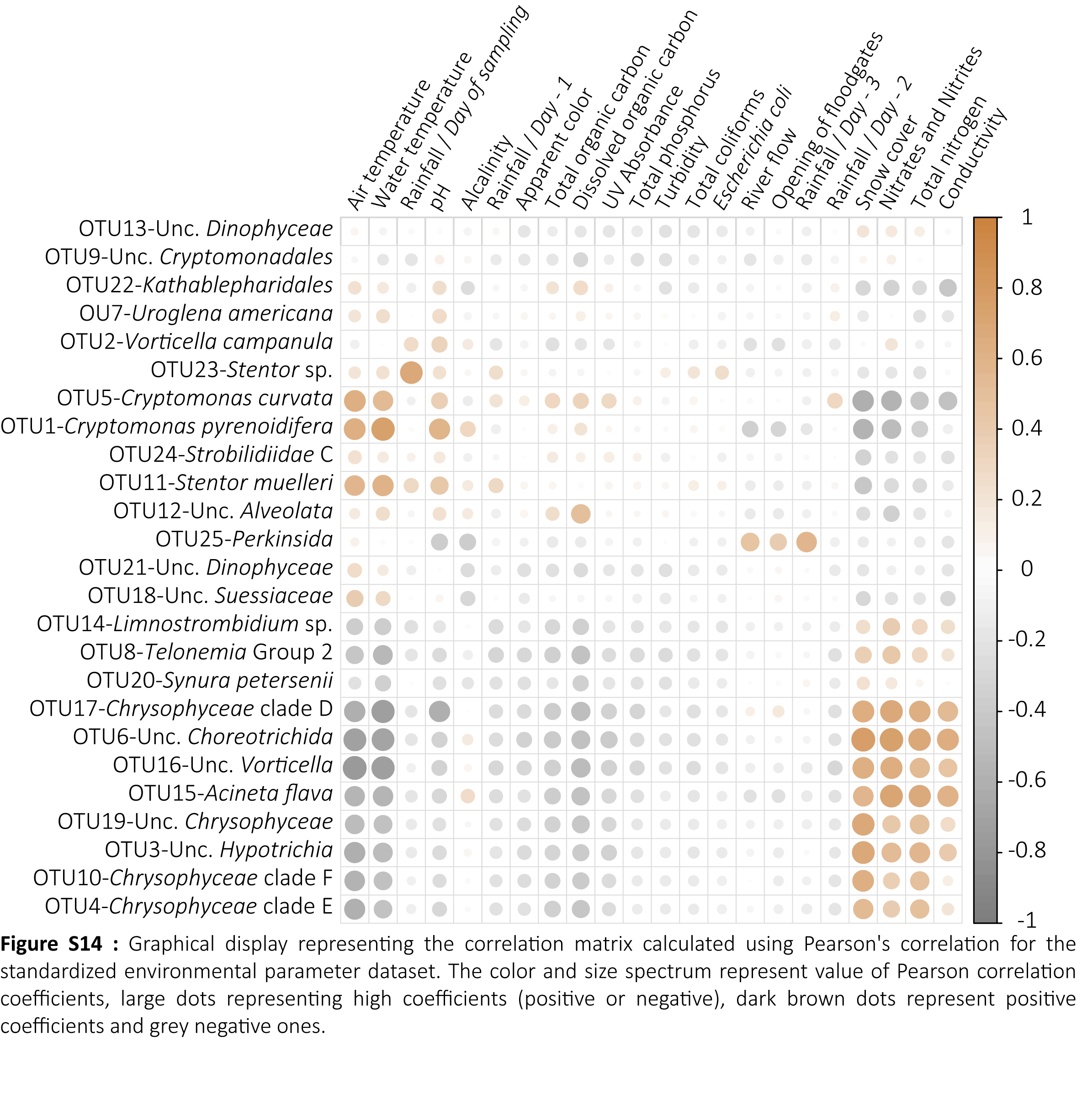

Supplement: Supplementary file 14 [file Image_14.tif]
